# Changes in children’s and adolescents’ dietary intake after the implementation of Chile’s law of food labeling, advertising and sales in schools: a longitudinal study

**DOI:** 10.1186/s12966-023-01445-x

**Published:** 2023-04-04

**Authors:** Gabriela Fretes, Camila Corvalán, Marcela Reyes, Lindsey Smith Taillie, Christina D. Economos, Norbert L.W. Wilson, Sean B. Cash

**Affiliations:** 1 International Food Policy Research Institute (IFPRI), 1201 Eye St NW, Washington, DC USA; 2 Instituto de Nutrición y Tecnología de los Alimentos (INTA), Universidad de Chile, Santiago, Chile; 3 Department of Nutrition, Gillings School of Global Public Health, University of North Carolina, Chapel Hill, NC USA; 4 Global Food Research Program, Carolina Population Center, Chapel Hill, NC USA; 5 Friedman School of Nutrition Science and Policy, Tufts University, Boston, MA USA; 6 Duke Divinity School, Sanford School of Public Policy, and World Food Policy Center, Duke University, Durham, NC USA

**Keywords:** Food labeling, Food environment, School food, Children, Adolescents

## Abstract

**Background:**

In June 2016, a comprehensive food policy was implemented in Chile that included front-of-package warning labels on key nutrients of concern (total sugars, added saturated fats, sodium, and calories), child-directed food advertisement bans, and school regulations. The policy was implemented in 3 phases from 2016 to 2019 and the primary objective was to improve children’s food environments. This study’s objective was to assess changes in child and adolescent intake of key nutrients of concern (total sugars, saturated fats, and sodium) at school after the initial implementation of Chile’s Law of Food Labeling and Advertisement.

**Methods:**

Longitudinal study of 349 children from the Food Environment Chilean Cohort (FECHIC) and 294 adolescents from the Growth and Obesity Cohort Study (GOCS). Data were from single 24-hour dietary recalls collected from 2016 to 2019. Fixed-effects models stratified by school, home, and other locations compared nutrient consumption in each year to consumption at the pre-policy 2016 baseline. Nutrient intakes are expressed as percent of total energy.

**Results:**

Compared to 2016 (pre-policy), total sugars consumed by children at school decreased 4.5 [-8.0, -0.9] percentage points (pp) and 11.8 [-15.4, -8.3] pp in 2018 and 2019 respectively. In 2019, children’s saturated fats and sodium intake at school also decreased (1.1 [-1.9, -0.2] pp and 10.3 [-18.1, -2.5] mg/100 kcal respectively). Likewise, in adolescents, total sugars and saturated fats consumed at school decreased in 2018 (5.3 [-8.4, -2.2] pp and 1.5 [-2.7, -0.3] pp respectively). However, consumption of key nutrients of concern at other locations increased after implementation of the policy.

**Conclusions:**

After initial implementation of Chile’s Labeling Law, intake of most key nutrients of concern significantly declined at school. However, we found evidence of compensatory behavior in out-of-school settings. Further research is needed to evaluate what other actions are needed to impact overall diets in the long term both at schools and out of school.

**Supplementary Information:**

The online version contains supplementary material available at 10.1186/s12966-023-01445-x.

## Background

Childhood obesity prevalence is a public health problem in Chile where 25.4% of school children live with obesity [1]. Many factors contribute to obesity, including diets, eating behaviors, and the food environment [2]. In Chile, children from low- to middle-income backgrounds are consuming over a quarter of their daily calories from snacks high in energy, total sugars, sodium, and/or saturated fat [3], and ultra-processed foods are the main source of energy [4]. Although most foods are consumed at home, foods consumed away from home tend to have higher caloric densities than foods consumed at home [5]. Considering the different food environments in which children, adolescents, and their caregivers make food choices that can impact their diets, the need for policy actions that improve the healthfulness of the food environment and guide people towards healthier food behaviors remains high [6, 7].

In 2016, the Chilean government implemented a comprehensive set of obesity prevention policies with the aim of improving children’s food environments [8]. Chile’s Law of Food Labeling and Advertising (LFLA) includes: (a) mandatory front-of-package (FOP) warning labels on packaged foods, (b) restrictions on all forms of food marketing directed to children < 14 years, and (c) school regulations at the pre-school, elementary and high school levels [9]. The LFLA required that food manufacturers must place up to four FOP labels on packaged foods or beverages that exceed cutoffs for added total sugars, saturated fats, sodium, and/or energy (nutrients of concern from hereafter). The FOP labels are black-and-white octagons that read “high-in” the nutrient of concern [8]. The government rolled out the policy in three annual phases, starting in 2016, from more flexible to more stringent cutoffs for defining regulated food and beverages [10].

Of particular interest for this study are school regulations. Chile’s LFLA mandates that foods and beverages with at least one FOP cannot be sold, promoted, or marketed inside schools (e.g., school kiosks, cafeteria, events). However, the regulation did not cover neighborhoods surrounding schools [11]. As initially drafted, the regulation did include these neighborhoods, but this stipulation was withdrawn from the final version of the LFLA enacted in 2012 [9]. Additionally, food and beverages with FOP warning labels cannot be offered as part of the school meals program or as free samples or gifts. The LFLA is unique because it includes a package of interventions covering several aspects of the school food environment, such as the availability of foods for sale inside schools, school meals program standards, and restrictions on food marketing directed to children. School food policies that target foods provided or available for sale in schools can improve children’s health and nutrition given the near universal access to food the setting provides to children and the time children spend in school [12, 13]. Researchers have found that school policies or guidelines that promote healthier school food environments significantly improved children’s dietary outcomes [12, 14–16]. Early evaluations of the Chilean LFLA on the availability of foods and beverages with FOP warning labels sold at school kiosks in Santiago found a significant decline in the availability of products with FOP warning labels [14]. However, research has not shown if these changes in the school food environment influenced children’s and adolescents’ diets at school, nor the impact of changes in the school meals program.

Evaluations regarding the initial FOP warning label (a) and marketing restrictions (b) components of the LFLA have shown positive results and include a decline in household purchases of nutrients of concern [17], a decrease in household sugar sweetened beverages purchases [18], a decline in youth exposure to food advertising [19, 20], and a good understanding of the meaning of the warning label as reported by mothers of young children and adolescents [21]. Evaluations of the school regulation component of the policy (c) focusing on behavior change at the individual level, however, are still lacking. As such, evidence is needed to understand changes in children’s and adolescents’ dietary intake at school after the implementation of Chile’s LFLA. In addition, evaluating changes in non-school settings such as home and other locations is important to understand how children navigate different food environments [7]. This study’s objective was to assess children’s and adolescents’ changes in dietary intake of key nutrients of concern (total sugars, added saturated fats, and sodium) at school and in non-school settings after implementation of the Chilean LFLA. We hypothesize that after implementation of the regulation, (a) children and adolescents eat fewer key nutrients of concern at school and that (b) this decrease is more significant at school than in other settings.

## Methods

### Study population and setting

The data used in this study are drawn from a longitudinal study of children (4-6y) from the Food Environment Chilean Cohort (FECHIC) and adolescents (12-14y) from the Growth and Obesity Cohort Study (GOCS) [3, 5]. Participants were recruited from low and middle-income neighborhoods in southeastern Santiago, Chile starting in 2016 (FECHIC) and 2006 (GOCS). Recruitment was facilitated through collaboration with the Chilean National Association of Day Care Centers and the National School Assistance and Scholarship Board. Additional details about inclusion and exclusion criteria as well as recruitment procedures of both cohorts have been described elsewhere [3, 5].

### Data collection

In this study, we used longitudinal data collected in 2016, 2017, 2018 (children & adolescents) and 2019 (children only). Data available for each participant included one 24-hour dietary recall (plus a second 24-hour recall for a random subset of the sample: ~20%), anthropometric measures for participants and their mothers, and sociodemographic data. For this study, the total analytical sample included 349 children (Figure S1) and 294 adolescents (Figure S2) who reported a first 24-hour dietary recall from January to July of each study year. We excluded recalls where consumption was reported as unusual (i.e., sick, birthday, holiday). Participants who did not have all 4 (FECHIC) or 3 (GOCS) study years of dietary and/or anthropometric data were excluded as well. For more information about the characteristics of participants included and not included in the analytical sample, see Additional file 1 Table S1. Although our study’s main focus is on schools, we also investigated intake at home and at other locations. Therefore, we considered 24-hour dietary recalls from all days of the week to be able to capture intake across locations.

Chile’s LFLA had a staggered implementation starting in June 2016 with the first phase, followed by the second phase in June 2018 and the third phase in June 2019. Baseline dietary data (pre-policy) were collected over weekdays and weekends through in-person interviews conducted by trained dietitians from January to July 2016. Subsequent waves of data were collected during the same period for consistency. Data collection was conducted with SER-24 software following the United States Department of Agriculture (USDA) standardized multi-pass method [22]. For nutritional information, we used the USDA Food Composition Database, which remained consistent over the whole time period, and linked this to the dietary intake data in each year [23]. Because this nutrient data remains constant over time, it does not account for reformulation, new product entry, or product exit that occurred in response to the Chilean LFLA [24] and thus reflects only behavioral changes observed during the implementation of the LFLA (e.g., children’s consumption of different types or amounts of food).

To help participants identify serving sizes of common food and beverages in the Chilean context, dietitians used the Photographic Atlas of Chilean Foods and Typical Preparations [25]. Recalls for children were completed by the primary caregiver (in > 90% of the cases, the mother) and adolescents answered on their own. In the interview, participants were asked about the location at which they consumed each food and beverage reported in the 24-hour dietary recall. Based on previous research exploring consumption of key nutrients of concern by eating location [5], we identified three categories: (a) home, by which we mean the participant’s personal residence; (b) school; and (c) other locations, including the homes of friends or other family members, food courts, cinemas, restaurants, etc.

Trained dietitians collected anthropometric measurements for children, adolescents, and their mothers (weight and height) annually beginning in 2016, using standardized techniques as described elsewhere [26]. Maternal body mass index (BMI) was calculated as weight in kilograms divided by the square of height in meters (kg/m^2^). For children and adolescents, BMI for age z-scores were estimated based on the World Health Organization (WHO) 2007 growth standards [27]. Sociodemographic data included age and biological sex of children and adolescents, mother’s age, maternal educational level, and marital status of mother.

### Outcomes

We focused the analyses on the key nutrients of concern that were subject to Chile’s LFLA reported in the first 24-hour dietary recall. Because participants from both cohorts are growing, we used a percent of energy outcome since it allows for total calories to increase as the participants’ energy requirements increase. Primary outcomes were changes in the percent of energy from key nutrients of concern consumed at school: (a) mean percent of energy from total sugars from total energy consumption at school, (b) mean percent of energy from saturated fats from total energy consumption at school, and (c) mean consumption of sodium (mg/100 kcal) at school. Secondary outcomes were changes in the percent of energy from each nutrient of concern consumed at home and other locations.

### Independent variables

Chile’s LFLA implementation, rolled out in three annual phases, was represented as a set of dummy variables for time with baseline at 2016 (pre-policy). With these variables, we aimed to capture both time and the impact of the first stage (2016) of the roll-out for children and adolescents; the second stage (2018) included only children.

### Covariates

Maternal educational level, child’s/adolescents’ BMI z-score, and weekday were included as covariates for adjusted analyses. Weekday was included as covariate in home and other locations models. We additionally included as covariates the consumption of nutrients of concern at other locations different from the outcome location.

### Statistical analyses

The hypotheses, variables of interest, and analysis were registered prior to analysis with the Open Science Framework (https://osf.io/bhscu).

All analyses were performed using Stata version 16.1 (College Station, TX, USA). We described the sociodemographic and anthropometric characteristics and consumption of key nutrients of concern of participants at baseline (pre-policy; 2016). Descriptive statistics such as mean, median, variance, maximum, and minimum were calculated for each variable and outliers were identified.

Unadjusted and adjusted fixed-effects models compared nutrient consumption in each study year to consumption at baseline by eating location. For each outcome, we calculated 95% confidence intervals (CIs) to evaluate statistical significance. Models included clustering for repeated measures at the individual level. The model specification for nutrient consumption took the following general form:

$$\begin{aligned}{nutlocation}_{it}&= {\beta }_{0}+ {\delta }_{1}{T}_{1}+ . . {\delta }_{t}{T}_{t}\\&\quad+{\boldsymbol{\beta}\mathbf{X}}_{\mathbf{i}\mathbf{t}} + {\alpha }_{i}+{\epsilon }_{it}, t=1,\dots ,T,\end{aligned}$$

where ${nutlocation}_{it}$ is a continuous measure of the percentage of energy from each nutrient for child and adolescent *i* in year *t* consumed at outcome location; ${\delta }_{t}$ is the coefficient of interest that shows the mean change from baseline; ${T}_{t}$ is a dummy variable for time (so we have t-1 time periods); $\boldsymbol{\beta}$ is the coefficient of the covariates; ${\mathbf{X}}_{\mathbf{i}\mathbf{t}}$ is a vector of covariates that includes maternal educational level, child/adolescent BMI z score, consumption at locations different from outcome, and weekday; ${\alpha }_{i}$ denotes the unobserved time-invariant individual factors; and ${\epsilon }_{it}$ is the error term, assumed to be uncorrelated to all the explanatory variables across all time periods.

### Sensitivity analyses

In addition to the unadjusted and adjusted fixed effects models, we conducted analyses excluding outliers (99th percentile) based on implausible values, analyses considering participants who did not have all study years of dietary and/or anthropometric data (pooled analysis), analyses considering data collected only on weekdays, and adjusted mixed effects models.

## Results

### Participant characteristics

Table 1 shows participants’ demographic, anthropometric, and nutrition characteristics at baseline. Children were on average 4.7 years old (SD 0.5) and 49.7% were female. The mean age of adolescents was 13.6 years (SD 0.4) and 43.2% were female. We found a similar overweight and obesity prevalence in children and adolescents with about 45% of our sample being affected.

**Table 1:** Characteristics of participants at baseline, 2016

	Children (n = 349)		Adolescents (n = 294)	
	Mean	SD	Mean	SD
*Child and adolescent characteristics*				
Age (years)	4.7	0.5	13.6	0.4
Female (%)	49.7		43.2	
BMI for age (z-score)	0.9	1.2	0.8	1.1
Weight status (%)				
Healthy	56.0		56.5	
Overweight	30.5		28.6	
Obesity	13.5		15.0	
*Nutrition*				
Energy (kcal/d)	1,220.3	363.8	1,837.3	598.2
Total sugars (g/d)	86.3	34.2	100.2	52.6
Total sugars (% daily energy)	28.4	7.8	21.7	8.7
Saturated fats (g/d)	13.3	6.0	20.3	10.8
Saturated fats (% daily energy)	9.8	3.2	9.7	3.1
Sodium (mg/d)	1,429.6	599.2	2,360.1	1,009.3
Sodium (mg/100 kcal)	118.8	41.1	131.3	45.5
*Children and adolescents reporting locations (%)*				
Home	98.9		98.6	
School	67.3		72.0	
Other	31.8		29.3	
*Children and adolescents reporting days (%)*				
Weekday	85.7		84.3	
Weekend	14.3		15.7	
*Mothers’ characteristics*				
Age (years)	31.7	6.8	40.7	7.6
Education level (%)				
Less than high school	18.1		35.4	
High school	46.7		42.8	
More than high school	35.2		21.8	
BMI (kg/m2)	28.7	5.9	29.9	5.3
Weight status (%)				
Healthy	29.3		18.4	
Overweight	34.9		35.5	
Obesity	35.8		46.2	
Married or living with partner (%)	52.9		47	

Note: Children are from the Food Environment Chilean Cohort (FECHIC). Adolescents are from the Growth and Obesity Cohort Study (GOCS). BMI z-scores were used to define children’s and adolescents’ weight status categories as healthy ( < = 1SD), overweight (> 1SD to < = 2 SD), and obesity (> 2SD). Underweight (<-2SD) was not identified in this sample. BMI was used to define mother’s weight status categories as healthy (18.5–24.9 kg/m2), overweight (25-29.9 kg/m2), and obesity ( > = 30 kg/m2)

**Fig. 1:**
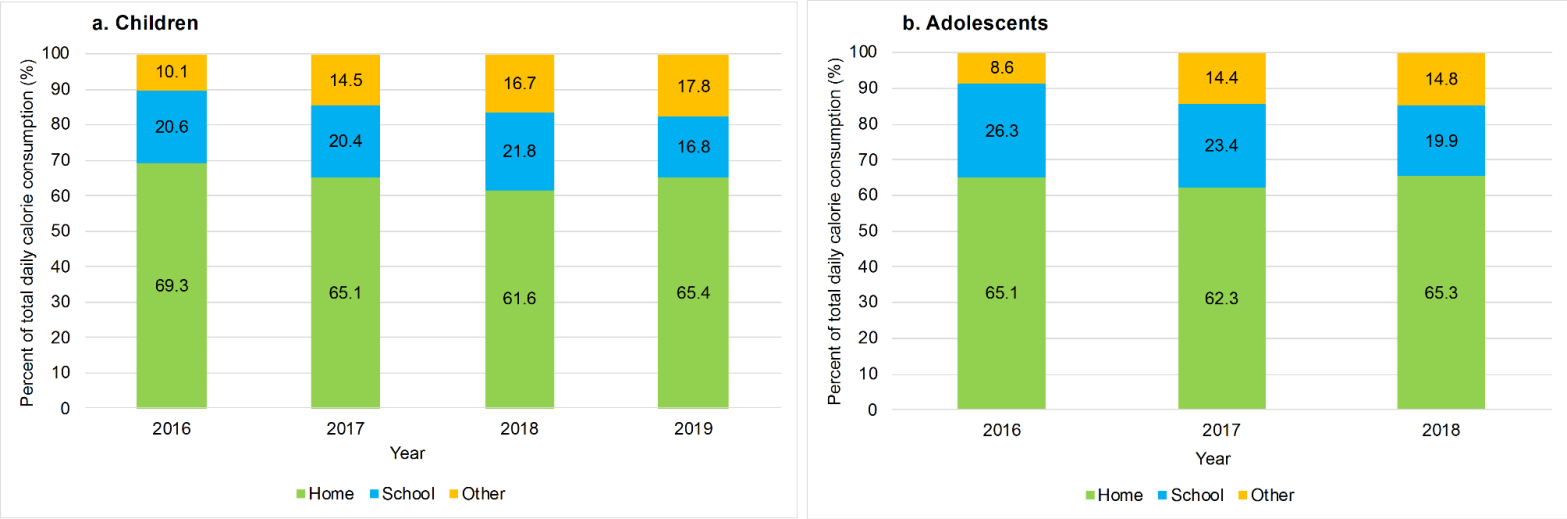
Share of total daily energy consumption in children and adolescents by eating location Children are from the Food Environment Chilean Cohort (FECHIC) 2016–2019 (n = 349) and adolescents are from the Growth and Obesity Cohort Study (GOCS) 2016–2018 (n = 294). Percentages were calculated by dividing calorie consumption at each eating location (home, school, other) by the total daily calorie consumption

About 70% of participants reported to eat at school on a given day and about 30% eat at other locations. At baseline, most of the daily energy consumption of children and adolescents came from foods consumed at home (69.3% and 65.1% respectively), followed by school (20.6% and 26.3% respectively) and other locations (10.1% and 8.6% respectively) (see Table S2 for participants’ characteristics in Year 1, 2 and 3 after policy). In Fig. 1 we observe that eating locations’ relative contributions to total daily energy intake changed over time, with a decrease in the percent of energy from home and school and an increase from other locations (see Table S3 for absolute values).

### Changes in children’s and adolescents’ percent of energy from total sugars, saturated fats, and sodium after Chile’s LFLA implementation by eating location

#### School

Children’s percent of energy from total sugars at school significantly decreased after policy implementation in 2018 and 2019 compared to 2016 (Table 2). The percentage of calories consumed at school from total sugars decreased 4.5 percentage points (pp) (95% CI -8.0, -0.9) in 2018 and 11.8 pp (95% CI -15.4, -8.3) in 2019 compared to 2016. Compared to 2016, the percentage of calories consumed at school from saturated fats and sodium also significantly decreased in 2019 (1.1 pp (95% CI -1.9, -0.2) and 10.3 milligrams/100 kcal (95% CI -18.1, -2.5) respectively).

**Table 2:** Changes in children’s percent of energy from total sugars, saturated fats, and sodium after Chile’s LFLA implementation by eating location

	Adjusted models Children (n = 349)				Unadjusted models Children (n = 349)			
**Year**	Baseline (95% CI)	Year 1 of Policy Absolute difference (95% CI)	Year 2 of Policy Absolute difference (95% CI)	Year 3 of Policy Absolute difference (95% CI)	Baseline (95% CI)	Year 1 of Policy Absolute difference (95% CI)	Year 2 of Policy Absolute difference (95% CI)	Year 3 of Policy Absolute difference (95% CI)
*School*								
Total sugars (%)	27.2 (24.5, 29.9)	-0.6 (-4.3, 3.2)	-4.5* (-8.0, -0.9)	-11.8* (-15.4, -8.3)	27.2 (24.5, 29.9)	-0.8 (-4.4, 2.9)	-3.9* (-7.3, -0.4)	-11.2* (-14.7, -7.6)
Saturated fats (%)	5.5 (4.9, 6.2)	-0.0 (-0.9, 0.8)	0.5 (-0.4, 1.4)	-1.1* (-1.9, -0.2)	5.5 (4.9, 6.2)	-0.1 (-0.9, 0.7)	0.6 (-0.3, 1.5)	-1.0* (-1.9, -0.2)
Sodium (mg/100 kcal)	50.3 (44.8, 55.7)	6.2 (-1.9, 14.4)	6.6 (-2.4, 15.6)	-10.3* (-18.1, -2.5)	50.3 (44.8, 55.7)	4.7 (-3.3, 12.8)	6.3 (-2.6, 15.2)	-10.9* (-18.6, -3.3)
*Home*								
Total sugars (%)	27.9 (26.7, 29.1)	-0.4 (-2.1, 1.3)	-2.3* (-3.9, -0.6)	-4.5* (-6.2, -2.8)	27.9 (26.7, 29.1)	-0.6 (-2.3, 1.2)	-2.2* (-3.8, -0.5)	-3.7* (-5.3, -2.0)
Saturated fats (%)	9.8 (9.3, 10.2)	0.0 (-0.6, 0.7)	0.0 (-0.6, 0.7)	0.1 (-0.6, 0.7)	9.8 (9.3, 10.2)	0.0 (-0.6, 0.6)	0.0 (-0.7, 0.7)	0.0 (-0.7, 0.7)
Sodium (mg/100 kcal)	127.2 (121.5, 132.8)	-8.1 (-18.0, 1.8)	-3.2 (-11.4, 4.8)	3.6 (-5.1, 12.4)	127.2 (121.5, 132.8)	-7.1 (-17.5, 3.2)	-3.1 (-11.0, 4.9)	1.3 (-7.6, 10.2)
*Other*								
Total sugars (%)	10.5 (8.5, 12.6)	4.3* (1.2, 7.4)	6.5* (3.1, 9.8)	5.8* (2.4, 9.2)	10.5 (8.5, 12.6)	4.4* (1.3, 7.4)	6.3* (3.0, 9.7)	5.9* (2.6, 9.2)
Saturated fats (%)	3.6 (2.8, 4.3)	1.7* (0.6, 2.8)	1.3* (0.3, 2.4)	1.2* (0.2, 2.2)	3.6 (2.8, 4.3)	1.7* (0.6, 2.8)	1.2* (0.2, 2.3)	1.1* (0.1, 2.1)
Sodium (mg/100 kcal)	34.1 (27.0, 41.2)	16.4* (5.5, 27.3)	19.7* (8.7, 30.7)	20.8* (9.7, 31.8)	34.1 (27.0, 41.2)	16.9* (6.1, 27.7)	17.3* (6.4, 28.3)	19.9* (8.9, 30.9)
*Overall*								
Total sugars (%)	28.4 (27.6, 29.3)	-0.9 (-2.1, 0.3)	-1.6* (-2.7, -0.6)	-2.4* (-3.6, -1.2)	28.4 (27.6, 29.3)	-1.1 (-2.2, 0.1)	-1.7* (-2.8, -0.6)	-2.4* (-3.6, -1.2)
Saturated fats (%)	9.8 (9.5, 10.1)	0.2 (-0.3, 0.7)	0.0 (-0.4, 0.5)	0.4 (-0.1, 0.9)	9.8 (9.5, 10.1)	0.3 (-0.2, 0.7)	0.0 (-0.4, 0.5)	0.4 (-0.1, 0.9)
Sodium (mg/100 kcal)	118.8 (114.5, 123.1)	-4.7 (-11.0, 1.6)	0.7 (-5.5, 6.9)	5.4 (-0.9, 11.6)	118.8 (114.5, 123.1)	-3.3 (-9.7, 3.1)	1.0 (-5.3, 7.3)	4.8 (-1.5, 11.2)

Note: Absolute difference is the difference between each year’s mean consumption of nutrients of concern after policy compared to baseline. Estimates were derived from fixed-effects models comparing nutrient’s consumption in each year (2017, 2018 and 2019) to consumption at baseline (2016). Covariates in adjusted models include maternal education level, child’s BMI z-score and weekday. Data are from the Food Environment Chilean Cohort (FECHIC). For total sugars and saturated fats, we calculated the percentage of energy that each of these nutrients contributed to the total daily energy consumption at each eating location (home, school and other). For sodium, we estimated the intake of sodium (mg) per 100 kcal. Consumption includes weekends. *p < 0.005

For school, home, and other locations analyses, we additionally controlled for other consumption locations (e.g., if outcome was sugars at school, we controlled for sugars at home and sugars at other locations). Weekday not included in school models because most of the school consumption is on weekdays (only two 24-hour dietary recalls from participants with all surveys were from weekends)

Similarly, adolescents’ percent of energy from total sugars and saturated fats at school significantly decreased after policy implementation in 2018 compared to 2016 (Table 3). Of calories consumed at school, in 2018, adolescents consumed 5.3 pp (95% CI -8.4, -2.2) fewer total sugars than in 2016. In addition, adolescents’ percent of energy from saturated fats dropped 1.5 pp (95% CI -2.7, -0.3) in 2018 compared to 2016. We found no changes in adolescents’ sodium consumption at school.

**Table 3:** Changes in adolescents’ percent of energy from total sugars, saturated fats, and sodium after Chile’s LFLA implementation by eating location

	Adjusted models Adolescents (n = 294)			Unadjusted models Adolescents (n = 294)		
**Year**	Baseline (95% CI)	Year 1 of Policy Absolute difference (95% CI)	Year 2 of Policy Absolute difference (95% CI)	Baseline (95% CI)	Year 1 of Policy Absolute difference (95% CI)	Year 2 of Policy Absolute difference (95% CI)
*School*						
Total sugars (%)	19.5 (17.3, 21.6)	-2.7 (-5.5, 0.1)	-5.3* (-8.4, -2.2)	19.5 (17.3, 21.6)	-2.9* (-5.7, -0.1)	-5.3* (-8.2, -2.4)
Saturated fats (%)	7.6 (6.8, 8.4)	-0.1 (-1.2, 1.0)	-1.5* (-2.7, -0.3)	7.6 (6.8, 8.4)	-0.2 (-1.4, 0.9)	-1.6* (-2.7, -0.5)
Sodium (mg/100 kcal)	70.2 (62.6, 77.7)	2.6 (-8.4, 13.6)	-8.8 (-20.7, 3.1)	70.2 (62.6, 77.7)	-0.3 (-10.5, 9.9)	-10.7* (-21.7, 0.2)
*Home*						
Total sugars (%)	20.6 (19.3, 21.8)	-0.5 (-2.3, 1.3)	-0.4 (-2.4, 1.6)	20.6 (19.3, 21.8)	0.1 (-1.7, 1.9)	0.5 (-1.4, 2.4)
Saturated fats (%)	9.1 (8.6, 9.6)	0.1 (-0.5, 0.8)	-0.5 (-1.2, 0.1)	9.1 (8.6, 9.6)	0.1 (-0.6, 0.8)	-0.6 (-1.3, 0.0)
Sodium (mg/100 kcal)	143.7 (136.3, 151.1)	-4.7 (-15.4, 5.9)	-3.3 (-14.9, 8.3)	143.7 (136.3, 151.1)	-6.3 (-16.5, 4.0)	-2.7 (-13.9, 8.5)
*Other*						
Total sugars (%)	9.5 (7.3, 11.8)	4.2* (0.9, 7.6)	2.5 (-0.8, 5.8)	9.5 (7.3, 11.8)	4.7* (1.3, 8.1)	2.4 (-0.7, 5.4)
Saturated fats (%)	2.8 (2.2, 3.5)	1.2* (0.3, 2.1)	1.1* (0.1, 2.1)	2.8 (2.2 ,3.5)	1.2* (0.3, 2.1)	0.9* (0.0, 1.9)
Sodium (mg/100 kcal)	31.2 (24.1, 38.4)	20.7* (9.8, 31.6)	20.2* (7.9, 32.4)	31.2 (24.1, 38.4)	20.1* (9.8 ,30.5)	17.8* (6.5, 29.1)
*Overall*						
Total sugars (%)	21.6 (20.7, 22.7)	0.1 (-1.2, 1.3)	0.4 (-1.0, 1.8)	21.6 (20.7, 22.7)	0.5 (-0.8, 1.7)	0.8 (-0.5, 2.2)
Saturated fats (%)	9.7 (9.4 ,10.1)	0.2 (-0.3, 0.7)	-0.2 (-0.7, 0.3)	9.7 (9.4, 10.1)	0.1 (-0.4, 0.6)	-0.3 (-0.8, 0.2)
Sodium (mg/100 kcal)	131.3 (126.0, 136.5)	1.8 (-5.4, 9.1)	8.4* (0.5, 16.3)	131.3 (126.0, 136.5)	1.8 (-5.5, 9.1)	8.1* (0.5, 15.6)

Note: Absolute difference is the difference between each year’s mean consumption of nutrients of concern after policy compared to baseline. Estimates were derived from fixed-effects models comparing nutrient’s consumption in each year (2017 and 2018) to consumption at baseline (2016). Covariates in adjusted models include maternal education level, child’s BMI z-score and weekday. Data are from the Growth and Obesity Cohort Study (GOCS). For total sugars and saturated fats, we calculated the percentage of energy that each of these nutrients contributed to the total daily calorie consumption at each eating location (home, school and other). For sodium, we estimated the intake of sodium (mg) per 100 calories. Consumption includes weekends. *p < 0.005

For school, home, and other locations analyses, we additionally controlled for other consumption locations (e.g., if outcome was sugars at school, we controlled for sugars at home and sugars at other locations). Weekday not included in school models because most of the school consumption is on weekdays (only four 24-hour dietary recalls from participants with all surveys were from weekends)

In both cohorts, we observed that the magnitude of the effect at school increased over time as the thresholds for defining regulated foods and beverages became more stringent.

#### Home

Following the same trend as school consumption, children’s percent of energy from total sugars at home significantly decreased in 2018 and 2019 compared to 2016 (Table 2). Of calories consumed at home, in 2018, children consumed 2.3 pp (95% CI -3.9, -0.6) less total sugar than in 2016. In 2019, the decrease was greater with a 4.5 pp (95% CI -6.2, -2.8) difference compared to 2016. We found no changes in children’s saturated fats and sodium consumption at home in the study years.

For adolescents, we found no changes in the percentage of calories from total sugars, saturated fats, and sodium consumed at home (Table 3).

#### Other locations

For children, we found that the percentage of calories from total sugars, saturated fats, and sodium consumed at other locations significantly increased in 2017, 2018, and 2019 compared to 2016 (Table 2). In 2017, children consumed 4.3 pp (95% CI 1.2, -7.4) more total sugars, 1.7 pp (95% CI 0.6, 2.8) more saturated fats, and 16.4 milligrams/100 kcal (95% CI 5.5, 27.3) more sodium at other locations compared to 2016. Differences in consumption in subsequent years can be found in Table 2.

Likewise, we observed a significant increase in adolescents’ percent of energy from total sugars (2017: 4.2 pp, 95% CI 0.9, 7.6), saturated fats (2017:1.2 pp, 95% CI 0.3, 2.1; 2018: 1.1 pp, 95% CI 0.1, 2.1), and sodium (2017: 20.7 mg/100 kcal, 95% CI 9.8, 31.6; 2018: 20.2 mg/100 kcal, 95% CI 7.9, 32.4) at other locations in 2017 and 2018 compared to 2016 (Table 3).

### Sensitivity analyses

Excluding outliers did not change our results from adjusted models with children’s data (Table S6). With adolescents, although the magnitude of estimates from adjusted models remained the same without outliers in most cases, we observed a significant decrease in the percentage of calories from total sugars consumed at school (-2.9 pp, 95% CI -5.5, -0.3) in 2017 compared to 2016. In addition, the increase in the percent of energy from saturated fats at other locations (0.8 pp, 95% CI -0.1, 1.7) in 2018 was not significant at 5%.

Including participants who did not have all study years of dietary and/or anthropometric data (no restriction of all years of data) in our analyses did not change our results substantially in terms of magnitude. We observed, however, some changes with the significance of sodium consumption results (Table S7). For instance, changes in children’s sodium consumption at school (-5.4 milligrams/100 calories, 95% CI -11.8, 1.1) and adolescents’ sodium consumption at school (-2.7 milligrams/100 calories, 95% CI -11.8, 6.5) were not significant at 5% in 2019 and 2018 respectively.

Excluding participants who reported intake on a weekend day in our analyses did not change our results considerably either (Table S8 and Table S9). We observed that changes in sodium consumption at school were significant at 5% in 2017 for children (10.2 milligrams/100 calories, 95% CI 0.5, 19.9) and adolescents (-15.2 milligrams/100 calories 95% CI -28.8, -1.7).

In the adjusted mixed effect models considering participants who did and did not have all study years, our results remained consistent in most of the cases. However, the significance of some coefficients changed (Table S10 and Table S11). For example, changes in sugar consumption at school were significant at 5% in 2017 for children (-2.6 pp, 95% CI -4.7, -0.5) and for adolescents (-1.8 pp, 95% CI -3.6, -0.1).

## Discussion

Children’s and adolescents’ percentage of calories consumed at school from total sugars, saturated fats, and sodium significantly declined after implementation of Chile’s LFLA. To our knowledge, this is the first study to evaluate longitudinal changes in children’s and adolescents’ consumption of key nutrients of concern at school after implementation of the LFLA. Results are consistent with the evidence that the regulated school food environment is protecting children and adolescents from exposure to foods and beverages high in sugar, saturated fats, and sodium, therefore impacting consumption of regulated nutrients consumed at school [12, 16, 28]. Our findings align with a study that evaluated the implementation of the Smart Snacks in School standards in the United States which found that children attending school in states with laws requiring the implementation of healthy food standards consumed fewer added sugars and solid fats compared to children from states with no such laws. However, authors found no difference in sodium consumption [28]. Our study also aligns with other studies conducted in Chile that show an improvement in the nutrient content of foods sold at school kiosks [14]. A unique feature of the school environment component of Chile’s LFLA is that it considers a combination of actions that results in improving the nutrient quality of both packaged and unpackaged foods, thus targeting both industry and behavioral components associated with healthier diets. Future studies should evaluate the contribution of reformulation of packaged foods to changes in dietary intake to separate individual from industry behavior change policy effects. Moreover, our results show that regulating school meals programs such as Chile’s LFLA has potential for greater impact, particularly among low-income children [21].

In our study, we found evidence of partial compensatory behavior with an increase in the percent of calories from nutrients of concern consumed at other locations (e.g., restaurants, street, on transportation). We believe that there are three central reasons for partial compensatory behavior at other eating locations. First, research has shown that the energy contribution of foods eaten away from home increases as children age [29]. Poti et al. described that daily energy intake from foods eaten away from home went from 29% at 2-6y to 37% at 13-18y in US children [29]. As children become more independent, they interact with different food environments, particularly restaurants and fast food outlets that usually offer foods that are high in calories, sodium, fats, and sugar [30]. In our study, we note that energy obtained from school became less important as a contributor to overall calories. Second, Chile’s LFLA did not include neighborhoods surrounding schools; thus, children and adolescents who attend schools that ban sales of food and beverages with FOP warning labels may look for other places like restaurants, corner stores, or other outlets to consume or buy foods they enjoy [31]. Lastly, some children and adolescents may be influenced positively by the policy while others will look for alternative outlets to obtain the prohibited products [32]. Future studies should identify “who reacts how” in order to identify factors that can be contributing to heterogeneity of policy effects.

To influence overall consumption of key nutrients of concern, our results highlight the importance of considering the different food environments with which children and adolescents interact during the day and not only single settings [33]. While there have been local efforts in some neighborhoods in Santiago to implement zoning policies in schools (e.g., Macul), little evidence exists on whether these are implemented and monitored. Further research is needed in the region to inform which interventions and policies can act in a synergistic way for improving children’s and adolescents’ food environments comprehensively [34]. Moreover, what other settings become relevant as children transition to adulthood – such as workplaces, colleges, and youth groups – is also a key question to take into consideration as these adolescents age.

Children’s and adolescents’ home environments are also crucial to helping shape healthier diets. We found a smaller but significant decline in children’s percent of calories from total sugars consumed at home. However, we found no change in adolescents’ total sugar consumption at home. This could be because parents still control a large part of what is purchased and brought into the home and therefore have control over children’s consumption within the home [35]. In addition, in a study by Correa et al., mothers reported that after implementing the regulation, children were acting as change agents in their homes, asking for healthier snacks [21]. Adolescents, on the other hand, were less likely to change behavior and were more skeptical about changes in the food environment [21]. Interventions targeted to adolescents using tools such as social media campaigns are needed to complement Chile’s policy and nudge adolescents toward healthier behaviors.

### Strengths and limitations

Among the strengths of this study are the use of a longitudinal design during a 2-year (adolescents) and 3-year follow-up (children) period after policy implementation which allowed us to examine changes in our study population. In addition, we had access to dietary data collected before policy implementation on the specific nutrients that were under the scope of the regulation.

This study also has limitations. Most notably (and inevitably), children and adolescents from each cohort got older during the study period. As children age, they naturally change their dietary behaviors. In our study, we could not disentangle age from policy effects with the available data, and unfortunately good comparison data on differences in dietary intake by age group for Chilean youth do not exist for the time period studied here. However, our design and research questions still allow us to infer changes by eating location and identify compensatory behavior across locations, independent from a child’s age. Our study included children living in low-to-middle-income neighborhoods in Santiago so results may not be generalizable to other regions of Chile or other socioeconomic groups. We used one single 24-hour dietary recall which may not be representative of children’s and adolescents’ usual dietary intake; however, it has been documented to be a good method to estimate mean intake at the group level. Our study only analyzed data at the nutrient level and did not include food-level analyses. This is an area for future study in order to have a better understanding of the food sources of the nutrients of concern in children’s and adolescents’ diets. Lastly, we included 24-hour dietary recalls reported on weekdays and weekends when the main focus of the study was on school environments; yet, our sensitivity analyses demonstrated that including only weekday reporting days did not change results interpretation.

## Conclusions

Our results suggest that a set of policy interventions at the school level, including restrictions on sales and marketing of foods and beverages high in sugars, sodium, and saturated fats, may be promising to improve children’s and adolescents’ diets. The impact of these policies can be enhanced by strategies implemented in out-of-school food environments that are important for children’s and adolescents’ diets. Future research should determine what other actions are needed to impact overall diets, to ensure healthy diets for children and adolescents both at school and outside of it. Our findings have implications for public health and food and nutrition policy because they show the significance of implementing regulations to improve the food environment to affect children’s and adolescents’ diets. Exposure to healthier foods at school and easy-to-understand information can contribute to shaping healthier behaviors during childhood and to the prevention of diet-related diseases.

## Electronic supplementary material

Below is the link to the electronic supplementary material.

**Additional File 1: Figure S1**. Flow diagram of data, Food Environment Chilean Cohort (FECHIC). **Figure S2**. Flow diagram of data, Growth and Obesity Cohort Study (GOCS). **Table S1**. Characteristics of participants at baseline (included vs not included in analytical sample). **Table S2**. Characteristics of participants at Year 1, 2 and 3 of policy, 2017- 2019. **Table S3**. Share of calories by eating location in children and adolescents, 2016-2019. **Table S4**. Changes in children’s percent of energy from total sugars, saturated fats, and sodium by eating location after Chile’s law implementation with covariate coefficients, 2016-2019. **Table S5**. Changes in adolescents’ percent of energy from total sugars, saturated fats, and sodium by eating location after Chile’s law implementation with covariate coefficients, 2016-2018. **Table S6**. Changes in the percent of energy from total sugars, saturated fats, and sodium in children and adolescents by eating location after Chile’s law implementation, 2016-2019 (without outliers >99th percentile). **Table S7**. Changes in the percent of energy from total sugars, saturated fats, and sodium in children and adolescents by eating location after Chile’s law implementation, 2016-2019 (pooled analyses). **Table S8**. Changes in children’s percent of energy from total sugars, saturated fats, and sodium after Chile’s LFLA implementation by eating location (only weekdays). **Table S9**. Changes in adolescents’ percent of energy from total sugars, saturated fats, and sodium after Chile’s LFLA implementation by eating location (only weekdays). **Table S10**. Changes in children’s percent of energy from total sugars, saturated fats, and sodium by eating location after Chile’s law implementation, 2016-2019 (mixed models). **Table S11**. Changes in adolescents’ percent of energy from total sugars, saturated fats, and sodium by eating location after Chile’s law implementation with covariate coefficients, 2016-2018 (mixed models)

**Additional File 2**. STROBE Statement Checklist of items that should be included in reports of observational studies.

## Data Availability

The datasets generated and/or analyzed during the current study are not publicly available but are available from the corresponding author on reasonable request.

## Abbreviations

FECHIC: Food Environment Chilean Cohort
GOCS: Growth Obesity Cohort Study
LFLA: Law of Food Labeling and Advertising
BMI: Body mass index
USDA: United States Department of Agriculture
WHO: World Health Organization
Kcal: kilocalories
pp: Percentage points
