# Supplementary material for: Changes in children’s and adolescents’ dietary intake after the implementation of Chile’s law of food labeling, advertising and sales in schools: a longitudinal study
Source: Int J Behav Nutr Phys Act. 2023 Apr 4;20:40. doi: 10.1186/s12966-023-01445-x (PMC10074676; doi:10.1186/s12966-023-01445-x)
Supplement: Supplementary file 1 — Additional File 1: Figure S1. Flow diagram of data, Food Environment Chilean Cohort (FECHIC). Figure S2. Flow diagram of data, Growth and Obesity Cohort Study (GOCS). Table S1. Characteristics of participants at baseline (included vs not included in analytical sample). Table S2. Characteristics of participants at Year 1, 2 and 3 of policy, 2017- 2019. Table S3. Share of calories by eating location in children and adolescents, 2016-2019. Table S4. Changes in children’s percent of energy from total sugars, saturated fats, and sodium by eating location after Chile’s law implementation with covariate coefficients, 2016-2019. Table S5. Changes in adolescents’ percent of energy from total sugars, saturated fats, and sodium by eating location after Chile’s law implementation with covariate coefficients, 2016-2018. Table S6. Changes in the percent of energy from total sugars, saturated fats, and sodium in children and adolescents by eating location after Chile’s law implementation, 2016-2019 (without outliers >99th percentile). Table S7. Changes in the percent of energy from total sugars, saturated fats, and sodium in children and adolescents by eating location after Chile’s law implementation, 2016-2019 (pooled analyses). Table S8. Changes in children’s percent of energy from total sugars, saturated fats, and sodium after Chile’s LFLA implementation by eating location (only weekdays). Table S9. Changes in adolescents’ percent of energy from total sugars, saturated fats, and sodium after Chile’s LFLA implementation by eating location (only weekdays). Table S10. Changes in children’s percent of energy from total sugars, saturated fats, and sodium by eating location after Chile’s law implementation, 2016-2019 (mixed models). Table S11. Changes in adolescents’ percent of energy from total sugars, saturated fats, and sodium by eating location after Chile’s law implementation with covariate coefficients, 2016-2018 (mixed models) [file 12966_2023_1445_MOESM1_ESM.pdf]

# Additional File 1

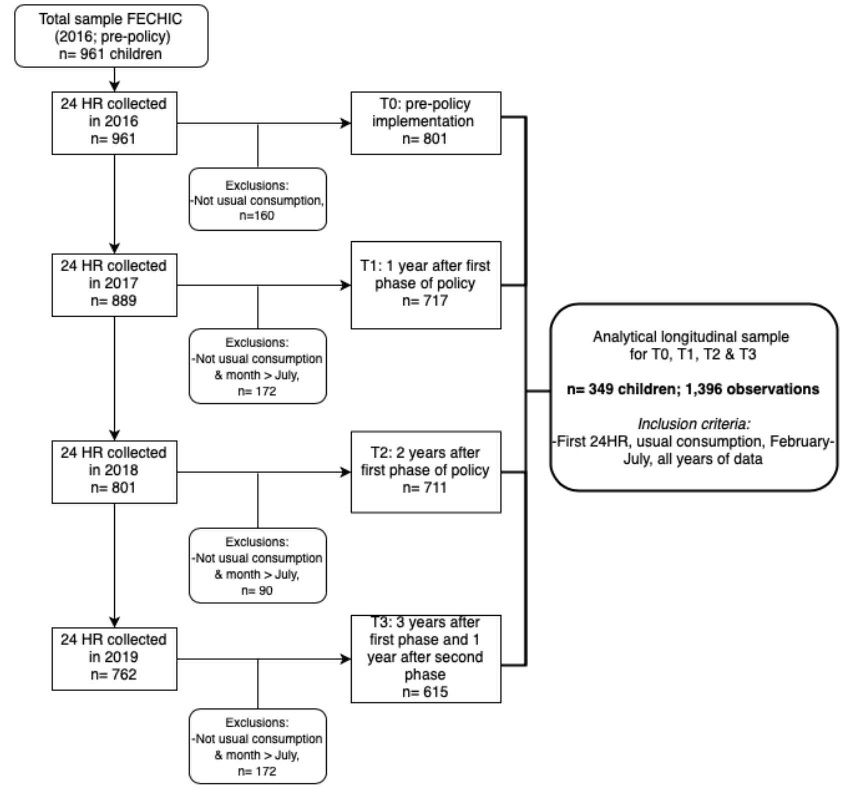


**Figure S1.** Flow diagram of data, Food Environment Chilean Cohort (FECHIC)

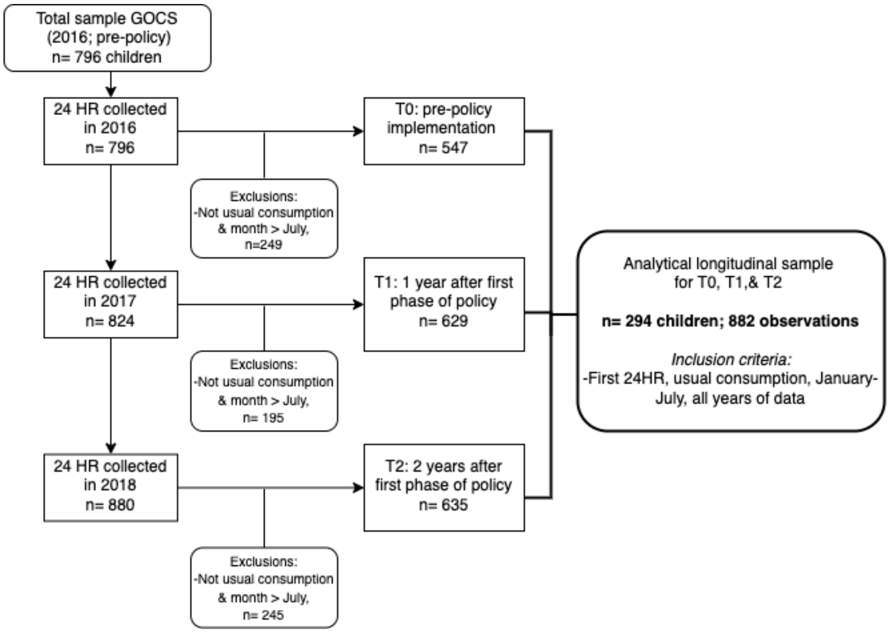


**Figure S2.** Flow diagram of data, Growth and Obesity Cohort Study (GOCS)

**Table S1. Characteristics of participants at baseline (included vs not included in analytical sample)**

| | Children | | Adolescents | |
|---|---|---|---|---|
| | Included in analytical sample (n = 349) | Not included in analytical sample (n = 612) | Included in analytical sample (n = 294) | Not included in analytical sample (n = 502) |
| *Child and adolescent characteristics* | 4.8 (0.5) | 4.8 (0.2) | 13.6 (0.4) | 13.7 (0.4) * |
| Age (years) | | | | |
| Female (%) | 49.7 | 52.9 | 43.2 | 53.2 |
| BMI for age (z-score) | 0.9 (1.2) | 1.1 (1.2) * | 0.8 (1.1) | 0.9 (1.1) * |
| Weight status (%) | | | | |
| Healthy | 56.0 | 51.6 * | 56.5 | 48.4 |
| Overweight | 30.5 | 27.7 * | 28.6 | 32.8 |
| Obesity | 13.5 | 20.7 * | 15.0 | 18.8 |
| *Nutrition* | | | | |
| Energy (kcal/d) | 1,220.3 (363.8) | 1,223.7 (415.5) | 1,837.3 (598.2) | 1,882.2 (704.3) |
| Total sugars (g/d) | 86.3 (34.2) | 88.3 (37.9) | 100.2 (52.6) | 103.9 (58.1) |
| Total sugars (% daily energy) | 28.4 (7.8) | 28.9 (8.9) | 21.7 (8.7) | 22.1 (8.8) |
| Saturated fats (g/d) | 13.3 (6.0) | 13.8 (7.1) | 20.3 (10.8) | 20.9 (10.6) |
| Saturated fats (% daily energy) | 9.8 (3.2) | 9.9 (3.4) | 9.7 (3.1) | 9.9 (3.1) |
| Sodium (mg/d) | 1,429.6 (599.2) | 1,479.8 (717.0) | 2,360.1 (1,009.3) | 2,436.2 (1,145.1) |
| Sodium (mg/100 kcal) | 118.8 (41.1) | 122.1 (51.2) | 131.3 (45.5) | 131.8 (46.9) |
| *Mothers' characteristics* | | | | |
| Age (years) | 31.7 (6.8) | 31.2 (6.6) | 40.7 (7.6) | 40.2 (7.2) |
| Education level (%) | | | | |
| Less than high school | 18.1 | 17.9 * | 35.4 | 25.7* |
| High school | 46.7 | 38.1 * | 42.8 | 46.0* |
| More than high school | 35.2 | 43.9 * | 21.8 | 28.3* |
| BMI (kg/m2) | 28.7 (5.9) | 28.8 (5.3) | 29.9 (5.3) | 29.3 (5.4) |
| Weight status (%) | | | | |
| Healthy | 29.3 | 25.4 | 18.4 | 19.3 |
| Overweight | 34.9 | 39.1 | 35.5 | 41.5 |
| Obesity | 35.8 | 35.5 | 46.2 | 39.2 |

Notes: *p<0.05. Standard deviations in parenthesis. For continuous variable group comparisons, we used ttests. For categorical variables, we used chi-square test. BMI z-scores were used to define children's and adolescents' weight status categories as healthy (<=1SD), overweight (>1SD to <=2 SD), and obesity (>2SD). Underweight (<-2SD) was not identified in this sample. BMI was used to define mother's weight status categories as healthy (18.5-24.9 kg/m2), overweight (25-29.9 kg/m2), and obesity (>=30 kg/m2).

**Table S2. Characteristics of participants at Year 1, 2 and 3 of policy, 2017- 2019**

| | **Children (n = 349)** | | | | | | **Adolescents (n = 294)** | | | |
|---|---|---|---|---|---|---|---|---|---|---|
| | Year 1 of Policy | | Year 2 of Policy | | Year 3 of Policy | | Year 1 of Policy | | Year 2 of Policy | |
| | Mean | SD | Mean | SD | Mean | SD | Mean | SD | Mean | SD |
| *Child and adolescent characteristics* | | | | | | | | | | |
| Age (years) | 5.7 | 0.4 | 6.6 | 0.4 | 7.5 | 0.4 | 14.5 | 0.4 | 15.5 | 0.4 |
| Female (%) | 49.7 | | 50.0 | | 49.9 | | 43.2 | | 43.2 | |
| BMI for age (z-score) | 0.9 | 1.2 | 0.9 | 1.2 | 1.1 | 1.3 | 0.7 | 1.1 | 0.6 | 1.1 |
| Weight status (%) | | | | | | | | | | |
| Healthy | 59.3 | | 54.8 | | 49.3 | | 61.4 | | 66.3 | |
| Overweight | 25.4 | | 25.8 | | 28.6 | | 24.9 | | 21.8 | |
| Obesity | 15.3 | | 19.4 | | 22.1 | | 13.6 | | 11.9 | |
| *Nutrition* | | | | | | | | | | |
| Energy (kcal/d) | 1,402.6 | 422.3 | 1,403.8 | 401.9 | 1,619.9 | 1,120.3 | 1,974.4 | 776.6 | 1847.6 | 716.8 |
| Total sugars (g/d) | 95.9 | 41.1 | 93.6 | 38.0 | 109.2 | 136.0 | 107.7 | 56.1 | 103.3 | 56.3 |
| Total sugars (% daily energy) | 27.4 | 8.5 | 26.7 | 8.0 | 26.1 | 9.3 | 22.1 | 8.4 | 22.5 | 8.9 |
| Saturated fats (g/d) | 15.9 | 8.3 | 15.6 | 7.5 | 18.9 | 18.6 | 22.2 | 13.1 | 19.7 | 10.9 |
| Saturated fats (% daily energy) | 10.0 | 4.9 | 9.8 | 3.1 | 10.2 | 3.7 | 9.8 | 3.3 | 9.4 | 3.2 |
| Sodium (mg/d) | 1,590.5 | 825.3 | 1,648.5 | 692.6 | 1,948.5 | 1,270.9 | 2,651.5 | 1,471.4 | 2,563.3 | 1,353.6 |
| Sodium (mg/100 kcal) | 115.5 | 50.4 | 119.8 | 42.9 | 123.6 | 42.9 | 133.0 | 44.2 | 139.3 | 48.5 |
| *Children and adolescents reporting locations (%)* | | | | | | | | | | |
| Home | 98.0 | | 97.4 | | 96.0 | | 97.3 | | 95.6 | |
| School | 67.9 | | 64.2 | | 46.7 | | 66.3 | | 54.1 | |
| Other | 47.3 | | 49.3 | | 48.4 | | 43.8 | | 39.5 | |
| *Children and adolescents reporting days (%)* | | | | | | | | | | |
| Weekday | 76.5 | | 84.0 | | 86.0 | | 80.0 | | 88.7 | |
| Weekend | 23.5 | | 16.0 | | 14.0 | | 20.0 | | 11.2 | |
| *Mothers' characteristics* | | | | | | | | | | |
| Age (years) | 32.5 | 6.8 | 33.5 | 6.9 | 34.4 | 6.8 | 41.5 | 7.6 | 42.4 | 7.4 |
| Education level (%) | | | | | | | | | | |
| Less than high school | 16.6 | | 16.1 | | 17.8 | | 32.9 | | 33.3 | |
| High school | 46.4 | | 47.8 | | 45.5 | | 47.3 | | 44.6 | |
| More than high school | 37.0 | | 36.1 | | 36.7 | | 19.7 | | 22.1 | |
| BMI (kg/m2) | 28.7 | 5.9 | 29.2 | 5.9 | 29.4 | 5.9 | 29.1 | 5.3 | 29.6 | 5.4 |
| Weight status (%) | | | | | | | | | | |
| Healthy | 26.4 | | 23.0 | | 23.4 | | 23.3 | | 16.7 | |
| Overweight | 39.8 | | 38.8 | | 37.8 | | 39.9 | | 41.4 | |
| Obesity | 33.8 | | 38.2 | | 38.7 | | 36.8 | | 41.9 | |
| Married or living with partner (%) | 52.1 | | 61.8 | | 47.8 | | 41.5 | | 43.9 | |

Note: Children are from the Food Environment Chilean Cohort (FECHIC). Adolescents are from the Growth and Obesity Cohort Study (GOCS). BMI z-scores were used to define children's and adolescents' weight status categories as healthy (<=1SD), overweight (>1SD to <=2 SD), and obesity (>2SD). Underweight (<-2SD) was not identified in this sample. BMI was used to define mother's weight status categories as healthy (18.5-24.9 kg/m2), overweight (25-29.9 kg/m2), and obesity (>=30 kg/m2).

**Table S3. Share of calories by eating location in children and adolescents, 2016-2019**

| | Children (n = 349) | | | | Adolescents (n = 294) | | |
|---|---|---|---|---|---|---|---|
| **Year** | Baseline (95% CI) | Year 1 of Policy (95% CI) | Year 2 of Policy (95% CI) | Year 3 of Policy (95% CI) | Baseline (95% CI) | Year 1 of Policy (95% CI) | Year 2 of Policy (95% CI) |
| *Percent of total daily energy* | | | | | | | |
| Home (%) | 69.3 (66.6 ,71.9) | 65.1 (62.2 , 67.9) | 61.6 (58.6 ,64.5) | 65.4 (62.2 , 68.6) | 65.1 (62.1 , 68.1) | 62.3 (59.1 , 65.4) | 65.3 (61.9 , 68.7) |
| School (%) | 20.6 (18.4 , 22.9) | 20.4 (18.2 , 22.6) | 21.8 (19.5 , 24.1) | 16.8 (14.5 , 19.1) | 26.3 (23.6 , 29.0) | 23.4 (20.8 , 25.9) | 19.9 (17.3 , 22.5) |
| Other (%) | 10.1 (8.0 ,12.2) | 14.5 (12.2 , 16.9) | 16.7 (14.1 , 19.3) | 17.8 (14.9 – 20.7) | 8.6 (6.5 , 10.6) | 14.4 (11.7 , 17.0) | 14.8 (11.9 ,17.7) |
| *Kcal absolute consumption* | | | | | | | |
| Home (mean) | 831.9 (792.1 , 871.7) | 901.6 (853.5 , 949.6) | 852.3 (804.9 , 899.8) | 1,063.8 (942.0 , 1,185.6) | 1,194.8 (1,123.4 , 1,226.2) | 1,206.4 (1,125.5 , 1,287.2) | 1,188.9 (1,104.4 ,1,273.3) |
| School (mean) | 255.1 (226.1 , 284.0) | 296.9 (261.8 , 332.1) | 316.7 (280.9 , 352.5) | 271.4 (231.4 , 311.4) | 467.6 (418.3 , 516.9) | 450.5 (395.9 , 505.1) | 366.8 (314.5 , 419.1) |
| Other (mean) | 133.3 (103.3 , 163.4) | 204.2 (168.7 , 239.6) | 234.8 (197.4 , 272.1) | 284.7 (233.2 , 336.1) | 174.9 (129.2 , 220.7) | 317.5 (246.9 , 388.1) | 291.9 (230.5 , 353.5) |

Note: Estimates were derived from fixed-effects models comparing nutrient's consumption in each year (2017, 2018 and 2019) to consumption at baseline (2016). Children are from the Food Environment Chilean Cohort (FECHIC) (n=349). Adolescents are from the Growth and Obesity Cohort Study (GOCS) (n=294). Percentages were calculated by dividing calorie consumption at each eating location (home, school, other) by the total daily calorie consumption.

**Table S4. Changes in children's percent of energy from total sugars, saturated fats, and sodium by eating location after Chile's law implementation with covariate coefficients, 2016-2019**

| **Outcome/ Eating location** | Total Sugars (%) B (95% CI) (n = 349) | Saturated fats (%) B (95% CI) (n = 349) | Sodium (mg/100 kcal) B (95% CI) (n = 349) |
|---|---|---|---|
| *School* | | | |
| Year after policy | | | |
| 1 | -0.6 (-4.3, 3.2) | -0.0 (-0.9 , 0.8) | 6.2 (-1.9 , 14.3) |
| 2 | -4.5* (-8.0 , -0.9) | 0.5 (-0.4 , 1.4) | 6.6 (-2.4 , 15.6) |
| 3 | -11.8* (-15.4 , -8.3) | -1.1* (-1.9 , -0.2) | -10.3* (-18.0 , -2.5) |
| Maternal education | | | |
| Less than high school | Ref | Ref | Ref |
| High school | -0.2 (-9.2 , 8.9) | -2.0 (-4.5 , 0.5) | -8.8 (-31.8 , 14.3) |
| More than high school | -5.2 (-16.7, 6.3) | -0.2 (-3.5 , 3.0) | 6.6 (-23.9 , 37.2) |
| Child BMI z-score | 0.9 (-2.3 , 4.2) | 0.4 (-0.4 , 1.2) | 1.8 (-5.7 , 9.3) |
| Nutrient consumption at home | -0.2* (-0.3 , -0.1) | -0.0 (-0.1 , 0.1) | 0.0 (-0.0 , 0.1) |
| Nutrient consumption at other locations | -0.0 (-0.1 , 0.0) | -0.0 (-0.1 , 0.0) | -0.1* (-0.1 , -0.0) |
| *Home* | | | |
| Year after policy | | | |
| 1 | -0.4 (-2.1 , 1.3) | 0.0 (-0.6 , 0.7) | -8.1 (-18.0 , 1.8) |
| 2 | -2.3* (-4.0 , -0.6) | 0.0 (-0.6 , 0.7) | -3.3 (-11.4 , 4.8) |
| 3 | -4.5* (-6.2 , -2.8) | 0.1 (-0.6 , 0.8) | 3.6 (-5.1 , 12.4) |
| Maternal education | | | |
| Less than high school | Ref | Ref | Ref |
| High school | -0.3 (-5.7 , 5.0) | 0.7 (-1.3 , 2.7) | 25.0* (1.2 , 48.8) |
| More than high school | -2.1 (-8.5 , 4.2) | 0.7 (-1.7 , 3.1) | 36.3* (6.6 , 65.9) |
| Child BMI z-score | 1.5* (0.2 , 2.9) | 0.0 (-0.7 , 0.7) | -4.1 (-15.7 , 7.6) |
| Weekday | 1.8 (-0.4 , 4.0) | -0.6 (-1.4 , 0.3) | -13.4* (-23.6 , -3.2) |
| Nutrient consumption at school | -0.1* (-0.1 , -0.0) | 0.0 (-0.1 , 0.1) | 0.1* (0.0 , 0.1) |
| Nutrient consumption at other locations | -0.0 (-0.0 , 0.0) | -0.0* (-0.1 , -0.0) | -0.1* (-0.1 , -0.0) |
| *Other* | | | |
| Year after policy | | | |
| 1 | 4.3* (1.2 , 7.4) | 1.7* (0.6 , 2.8) | 16.4* (5.5 , 27.3) |
| 2 | 6.5* (3.1 , 9.8) | 1.3* (0.3 , 2.4) | 19.7* (8.7 , 30.7) |
| 3 | 5.8* (2.5 , 9.2) | 1.2 *(0.2 , 2.2) | 20.8* (9.7 , 31.8) |
| Maternal education | | | |
| Less than high school | Ref | Ref | Ref |
| High school | -1.7 (-8.5 , 5.0) | -0.9 (-3.4 , 1.5) | -15.8 (-41.4 , 9.7) |
| More than high school | -0.8 (-10.3 , 8.7) | 0.2 (-3.4 , 3.8) | -18.9 (-58.9 , 21.1) |
| Child BMI z-score | 0.3 (-3.0 , 3.6) | -0.6 (-1.3 , 0.2) | -3.0 (-12.7 , 6.7) |
| Weekday | -2.3 (-6.5 , 1.9) | -0.3 (-1.5 , 0.8) | -13.5 (-28.7 , 1.8) |
| Nutrient consumption at school | -0.0 (-0.1 , 0.1) | -0.0 (-0.1 , 0.0) | -0.1 (-0.1 , 0.0) |
| Nutrient consumption at home | -0.0 (-0.1 , 0.1) | -0.1* (-0.2 , -0.0) | -0.1 (-0.2 , 0.0) |
| *Overall* | | | |
| Year after policy | | | |
| 1 | -0.9 (-2.1 , 0.3) | 0.2 (-0.3 , 0.7) | -4.7 (-11.0 , 1.6) |
| 2 | -1.6* (-2.7 , -0.6) | 0.0 (-0.4 , 0.5) | 0.7 (-5.5 , 6.9) |
| 3 | -2.4* (-3.6, -1.2) | 0.4 (-0.1 , 0.9) | 5.4 (-0.9 , 11.6) |

| | | | |
|---|---|---|---|
| Maternal education | | | |
| Less than high school | Ref | Ref | Ref |
| High school | -0.1 (-3.4 , 3.1) | -0.6 (-1.8 , 0.6) | 14.9 (-2.3 , 32.2) |
| More than high school | -0.9 (-4.8 , 2.9) | -0.3 (-1.8 , 1.2) | 19.1 (-3.1 , 41.3) |
| Child BMI z-score | 0.2 (-1.0 , 1.4) | 0.0 (-0.6 , 0.6) | -2.3 (-9.5 , 4.9) |
| Weekday | 2.0 (0.6 , 3.4) | -0.4 (-0.9 , 0.1) | -15.5* (-22.9 , -8.2) |

Note: Estimates were derived from fixed-effects models comparing nutrient's consumption in each year (2017, 2018 and 2019) to consumption at baseline (2016). Data are from the Food Environment Chilean Cohort (FECHIC). For total sugars and saturated fats, we calculated the percentage of energy that each of these nutrients contributed to the total daily energy consumption at each eating location (home, school and other). For sodium, we estimated the intake of sodium (mg) per 100 kcal. Consumption includes weekends. *p<0.005; confidence intervals in parenthesis.

**Table S5. Changes in adolescents' percent of energy from total sugars, saturated fats, and sodium by eating location after Chile's law implementation with covariate coefficients, 2016-2018**

| **Outcome/ Eating location** | Total Sugars (%) B (95% CI) (n = 294) | Saturated fats (%) B (95% CI) (n = 294) | Sodium (mg/100 kcal) B (95% CI) (n = 294) |
|---|---|---|---|
| *School* | | | |
| Year after policy | | | |
| 1 | -2.7 (-5.5 , 0.1) | -0.1 (-1.2 , 1.0) | 2.6 (-8.4 , 13.6) |
| 2 | -5.3* (-8.4 , -2.2) | -1.5* (-2.7 , 0.3) | -8.8 (1-20.7 , 3.1) |
| Maternal education | | | |
| Less than high school | Ref | Ref | Ref |
| High school | 3.1 (-3.5 , 9.7) | -0.0 (-2.5 , 2.5) | 3.4 (-21. 1 , 27.9) |
| More than high school | 2.9 (-3.6 , 9.6) | 0.6 (-2.1 , 3.3) | 11.2 (-16.8 , 39.1) |
| Adolescent BMI z-score | 0.3 (-5.3 , 5.9) | 0.5 (-1.2 , 2.3) | -1.3 (-20.1 , 17.4) |
| Nutrient consumption at home | 0.0 (-0.1 , 0.1) | -0.0 (-0.1 , 0.1) | -0.0 (-0.1 , 0.0) |
| Nutrient consumption at other locations | -0.0 (-0.1 , 0.1) | -0.0 (-0.1 , 0.1) | -0.1* (-0.2 , 0.0) |
| *Home* | | | |
| Year after policy | | | |
| 1 | -0.5 (-2.3 , 1.3) | 0.1 (-0.5 , 0.8) | -4.7 (-15.4 , 5.9) |
| 2 | -0.4 (-2.4 , 1.6) | -0.5 (-1.2 , 0.1) | -3.3 (-14.9 , 8.3) |
| Maternal education | | | |
| Less than high school | Ref | Ref | Ref |
| High school | 4.0 (-0.7 , 8.8) | 0.4 (-1.3 , 2.1) | 12.4 (-8.9 , 33.7) |
| More than high school | 3.4 (-1.5 , 8.2) | -0.1 (-1.9 , 1.7) | 20.7 (1.1 , 40.4) |
| Adolescent BMI z-score | -5.3* (-8.9 , 1.6) | 0.5 (-0.7 , 1.8) | 1.3 (-14.4 , 16.9) |
| Weekday | -0.3 (-3.1 , 2.5) | -0.2 (-1.2 , 0.8) | 13.4 (-0.2 , 27.0) |
| Nutrient consumption at school | 0.0 (-0.0 , 0.0) | 0.0 (-0.0 , 0.1) | -0.1 (-0.2 , 0.0) |
| Nutrient consumption at other locations | -0.0 (-0.1 , 0.0) | 0.0 (-0.1 , 0.1) | -0.0 (-0.1 , 0.1) |
| *Other* | | | |
| Year after policy | | | |
| 1 | 4.2* (0.9 , 7.6) | 1.2* (0.3 ,2.1) | 20.7* (9.8 , 31.6) |
| 2 | 2.5 (-0.8 , 5.8) | 1.1* (0.1 , 2.1) | 20.2* (7.9 – 32.4) |
| Maternal education | | | |
| Less than high school | Ref | Ref | Ref |
| High school | 5.0 (-3.1 , 13.1) | -1.3 (-3.6 , 0.9) | -13.4 (-41.1 , 14.4) |
| More than high school | -2.0 (-5.5 , 9.5) | -1.5 (-3.9 , 0.9) | -19.3 (-48.2 , 9.6) |
| Adolescent BMI z-score | -0.1 (-5.7 , 5.5) | 0.3 (-1.5 , 2.1) | 12.3 (-8.6 , 33.2) |
| Weekday | -4.9* (-9.7 , -0.2) | -1.7* (-3.1 , -0.3) | -22.6 (-40.7 - -4.5) |
| Nutrient consumption at school | -0.0 (-0.1 , 0.1) | 0.0 (-0.1 , 0.1) | -0.1 (-0.2 , 0.0) |
| Nutrient consumption at home | -0.1 (-0.3 , 0.0) | 0.0 (-0.1 , 0.1) | -0.0 (-0.2 , 0.1) |
| *Overall* | | | |
| Year after policy | | | |
| 1 | 0.1 (-1.2 , 1.3) | 0.2 (-0.3 , 0.7) | 1.8 (-5.4 , 9.1) |
| 2 | 0.4 (-1.0 , 1.8) | -0.2 (-0.7 , 0.3) | 8.4* (0.5 , 16.3) |
| Maternal education | | | |
| Less than high school | Ref | Ref | Ref |

| | | | |
|---|---|---|---|
| High school | 4.5* (1.3 , 7.7) | -0.2 (-1.5 , 1.1) | 4.4 (-13.6 , 22.3) |
| More than high school | 4.3* (0.9 , 7.7) | -0.5 (-1.9 , 1.0) | 9.2 (-6.9 , 25.3) |
| Adolescent BMI z-score | -2.1 (-4.4 , 0.2) | 0.6 (-0.3 , 1.6) | 1.2 (-9.4 , 11.8) |
| Weekday | -0.3 (-2.2 , 1.5) | 0.1 (-0.6 , 0.8) | -6.0 (-16.3 , 4.2) |

Note: Estimates were derived from fixed-effects models comparing nutrient's consumption in each year (2017 and 2018) to consumption at baseline (2016). Data are from the Growth Obesity Cohort Study (GOCS). For total sugars and saturated fats, we calculated the percentage of energy that each of these nutrients contributed to the total daily energy consumption at each eating location (home, school and other). For sodium, we estimated the intake of sodium (mg) per 100 kcal. Consumption includes weekends. *p<0.005; confidence intervals in parenthesis.

**Sensitivity analyses**

**Table S6. Changes in the percent of energy from total sugars, saturated fats, and sodium in children and adolescents by eating location after Chile's law implementation, 2016-2019 (without outliers >99th percentile)**

| | Adjusted models Children (n = 349) | | | | Adjusted models Adolescents (n = 294) | | |
|---|---|---|---|---|---|---|---|
| **Year** | Baseline (95% CI) | Year 1 of Policy Absolute difference (95% CI) | Year 2 of Policy Absolute difference (95% CI) | Year 3 of Policy Absolute difference (95% CI) | Baseline (95% CI) | Year 1 of Policy Absolute difference (95% CI) | Year 2 of Policy Absolute difference (95% CI) |
| *School* | | | | | | | |
| Total sugars (%) | 26.6 (24.0 , 29.3) | -0.5 (-4.2 , 3.2) | -4.6* (-8.0 , -1.2) | -11.6* (-15.1 , -8.1) | 18.9 (16.8 , 21.0) | -2.9* (-5.5 , -0.3) | -5.1* (-7.8 , -2.3) |
| Saturated fats (%) | 5.3 (4.7 , 5.9) | 0.0 (0.7 , 0.8) | 0.4 (-0.5 , 1.2) | -1.1* (-1.8 , 0.2) | 7.4 (6.7 , 8.2) | -0.3 (-1.4 , 0.7) | -1.6* (-2.7 , -0.5) |
| Sodium (mg/100 kcal) | 48.1 (43.2 , 52.9) | 5.4 (-1.5 , 12.4) | 4.9 (-2.9 , 12.9) | -8.7* (-16.0 , -1.4) | 65.6 (58.8 , 72.3) | -0.5 (-9.4 , 8.5) | -9.1 (-19.1 , 0.8) |
| *Home* | | | | | | | |
| Total sugars (%) | 27.4 (26.3 , 28.5) | -0.8 (-2.3 , 0.7) | -2.3* (-3.8 , -0.8) | -4.5* (-5.9 , -3.0) | 20.6 (13.4 , 21.9) | -0.3 (-1.9 , 1.4) | 0.1 (-1.7 , 1.8) |
| Saturated fats (%) | 9.7 (9.3 , 10.2) | -0.0 (-0.7 , 0.5) | -0.2 (-0.8 , 0.5) | -0.2 (-0.8 , 0.5) | 9.0 (8.6 , 9.4) | -0.1 (-0.7 , 0.6) | -0.5 (-1.1 , 0.1) |
| Sodium (mg/100 kcal) | 125.8 (120.5 , 131.1) | -10.3* (-17.1 , -3.6) | -3.7 (-11.4 , 3.9) | -0.3 (-8.2 , 7.6) | 139.2 (133.3 , 145.1) | -2.7 (-11.6 , 6.2) | -0.7 (-10.1 , 8.8) |
| *Other* | | | | | | | |
| Total sugars (%) | 10.1 (8.1 , 12.0) | 3.4* (0.5 , 6.2) | 4.9* (1.9 , 7.9) | 5.7* (2.5 , 8.9) | 9.2 (7.0 , 11.4) | 3.8* (0.6 , 6.9) | 2.2 (-0.7 , 5.1) |
| Saturated fats (%) | 3.2 (2.6 , 3.9) | 1.5* (0.5 , 2.5) | 1.3* (0.4 , 2.2) | 1.3* (0.4 , 2.2) | 2.8 (2.2 , 3.5) | 1.1* (0.3 , 2.0) | 0.8 (-0.1 , 1.7) |
| Sodium (mg/100 kcal) | 30.1 (24.2 , 36.1) | 15.7* (6.8 , 24.6) | 16.6* (8.1 , 25.1) | 20.4* (11.9 , 28.8) | 28.4 (21.9 , 34.8) | 20.4* (10.8 , 30.0) | 16.6* (6.4 , 26.7) |
| *Overall* | | | | | | | |
| Total sugars (%) | 28.4 (27.6 , 29.2) | -0.9 (-2.0 , 0.3) | -1.6* (-2.7 , -0.6) | -2.8* (-3.9 , -1.7) | 21.5 (20.5 , 22.4) | 0.1 (-1.3 , 1.3) | 0.3 (-1.1 , 1.6) |
| Saturated fats (%) | 9.7 (9.4 , 10.0) | 0.1 (-0.3 , 0.6) | 0.0 (-0.3 , 0.5) | 0.4 (-0.1 , 0.8) | 9.6 (9.3 , 9.9) | 0.1 (-0.4 , 0.6) | -0.2 (-0.7 , 0.3) |
| Sodium (mg/100 kcal) | 117.7 (113.7 , 121.8) | -5.5* (-10.5 , -0.4) | -1.4 (-7.1 , 4.2) | 5.4 (-0.6 , 11.4) | 129.4 (124.8 , 133.8) | 3.5 (-3.4 , 10.3) | 8.2* (1.17 , 15.3) |

Note: Absolute difference is the difference between each year's mean consumption of nutrients of concern after policy compared to baseline. Estimates were derived from fixed-effects models comparing nutrient's consumption in each year (2017, 2018 and 2019) to consumption at baseline (2016). Models excluded values over 99th percentile. Covariates in adjusted models include maternal education level, child's BMI z-score and weekday. Data are from the Food Environment Chilean Cohort (FECHIC) and the Growth and Obesity Cohort Study (GOCS). For total sugars and saturated fats, we calculated the percentage of energy that each of these nutrients contributed to the total daily energy consumption at each eating location (home, school and other). For sodium, we estimated the intake of sodium (mg) per 100 kcal. Consumption includes weekends. *p<0.005

For school analyses, we additionally controlled for other consumption locations (e.g., if outcome was sugars at school, we controlled for sugars at home and sugar at other locations). Weekday not included in school models because most of the school consumption is on weekdays.

**Table S7. Changes in the percent of energy from total sugars, saturated fats, and sodium in children and adolescents by eating location after Chile's law implementation, 2016-2019 (pooled analyses)**

| | **Adjusted models Children (n = 947)** | | | | **Adjusted models Adolescents (n = 885)** | | |
|---|---|---|---|---|---|---|---|
| **Year** | Baseline (95% CI) | Year 1 of Policy Absolute difference (95% CI) | Year 2 of Policy Absolute difference (95% CI) | Year 3 of Policy Absolute difference (95% CI) | Baseline (95% CI) | Year 1 of Policy Absolute difference (95% CI) | Year 2 of Policy Absolute difference (95% CI) |
| *School* | | | | | | | |
| Total sugars (%) | 27.5 | -1.9 | -4.9* | -11.2* | 18.1 | -2.1 | -4.7* |
| | (25.7 , 29.3) | (-4.5 , 0.8) | (-7.4 , -2.4) | (-13.9 , -8.5) | (16.5 , 19.8) | (-4.4 , 0.2) | (-7.1 , -2.2) |
| Saturated fats (%) | 5.5 | -0.1 | 0.5 | -0.7* | 7.1 | -0.2 | -1.1* |
| | (5.1 , 5.9) | (-0.7 , 0.5) | (-0.2 , 1.2) | (-1.4 , -0.0) | (6.5 , 7.7) | (-1.1 , 0.6) | (-2.0 , -0.2) |
| Sodium (mg/100 | 50.9 | 3.1 | 5.8 | -5.4 | 61.9 | 4.7 | -2.7 |
| kcal) | (47.2 , 54.6) | (-2.7 , 8.9) | (-0.5 , 12.1) | (-11.8 , 1.1) | (56.7 , 67.2) | (-3.6 , 13.1) | (-11.8 , 6.5) |
| *Home* | | | | | | | |
| Total sugars (%) | 27.4 | -0.7 | -1.9* | -4.4* | 20.1 | 0.4 | -0.6 |
| | (26.6 , 28.2) | (-1.9 , 0.5) | (-3.1 , -0.6) | (-5.6 , -3.1) | (19.2 , 21.0) | (-1.1 , 1.8) | (-2.3 , 1.0) |
| Saturated fats (%) | 10.1 | -0.1 | -0.2 | 0.0 | 9.1 | 0.3 | -0.3 |
| | (9.8 , 10.4) | (-0.6 , 0.4) | (-0.6 , 0.3) | (-0.5 , 0.6) | (8.8 , 9.5) | (-0.2 , 0.9) | (-0.9 , 0.3) |
| Sodium (mg/100 | 129.2 | -5.7 | -2.8 | 3.6 | 140.2 | 4.2 | 4.5 |
| kcal) | (125.2 , 133.3) | (-12.5 , 1.1) | (-8.8 , 3.2) | (-3.0 , 10.3) | (135.1 , 145.4) | (-6.7 , 15.2) | (-6.2 , 15.2) |
| *Other* | | | | | | | |
| Total sugars (%) | 11.9 | 4.2* | 5.0* | 3.9* | 9.4 | 3.4* | 2.3 |
| | (10.4 , 13.4) | (1.9 , 6.5) | (2.5 , 7.5) | (1.4 , 6.4) | (7.7 , 11.0) | (0.7 , 6.1) | (-0.4 , 4.9) |
| Saturated fats (%) | 3.4 | 1.4* | 1.5* | 1.3* | 3.0 | 1.2* | 1.1* |
| | (2.9 , 3.8) | (0.7 , 2.2) | (0.7 , 2.3) | (0.5 , 2.1) | (2.5 , 3.5) | (0.4 , 1.9) | (0.2 , 1.9) |
| Sodium (mg/100 | 33.7 | 15.6* | 18.6* | 17.8* | 33.8 | -60.0 | 29.7 |
| kcal) | (29.1 , 38.4) | (7.7 , 23.4) | (10.8 , 26.2) | (9.3 , 26.3) | (28.4 , 39.2) | (-207.6 , 87.5) | (-6.6 – 65.9) |
| *Overall* | | | | | | | |
| Total sugars (%) | 28.5 | -1.1* | -1.6* | -2.7* | 21.4 | 0.5 | -0.0 |
| | (27.9 , 29.1) | (-1.9 , -0.2) | (-2.4 , -0.8) | (-3.6 , -1.8) | (20.7 , 22.1) | (-4.9 , 1.6) | (-1.1 , 1.1) |
| Saturated fats (%) | 9.8 | 0.1 | 0.1 | 0.4* | 9.8 | 0.1 | -0.0 |
| | (9.5 , 10.0) | (-0.2 , 0.5) | (-0.2 , 0.4) | (0.0 , 0.8) | (9.5 , 10.1) | (-0.3 , 0.4) | (-0.5 , 0.4) |
| Sodium (mg/100 | 119.1 | -3.9 | 0.4 | 6.2* | 130.1 | 5.9 | 11.8* |
| kcal) | (116.1 , 122.2) | (-8.4 , 0.6) | (-4.1 , 4.9) | (1.2 , 11.1) | (126.3 , 133.7) | (-1.7 , 13.5) | (4.7 , 19.1) |

Note: Absolute difference is the difference between each year's mean consumption of nutrients of concern after policy compared to baseline. Estimates were derived from fixed-effects models comparing nutrient's consumption in each year (2017, 2018 and 2019) to consumption at baseline (2016). Covariates include maternal education level, child's BMI z-score and weekday. Data are from the Food Environment Chilean Cohort (FECHIC) and the Growth and Obesity Cohort Study (GOCS). For total sugars and saturated fats, we calculated the percentage of energy that each of these nutrients contributed to the total daily energy consumption at each eating location (home, school and other). For sodium, we estimated the intake of sodium (mg) per 100 kcal. Consumption includes weekends. *p<0.005
For school, home and other locations analyses, we additionally controlled for other consumption locations (e.g., if outcome was sugars at school, we controlled for sugars at home and sugars at other locations). Weekday not included in school models because most of the school consumption is on weekdays.

**Table S8. Changes in children's percent of energy from total sugars, saturated fats, and sodium after Chile's LFLA implementation by eating location (only weekdays)**

| | Adjusted models Children (n = 349) | | | | Unadjusted models Children (n = 349) | | | |
|---|---|---|---|---|---|---|---|---|
| **Year** | Baseline (95% CI) | Year 1 of Policy Absolute difference (95% CI) | Year 2 of Policy Absolute difference (95% CI) | Year 3 of Policy Absolute difference (95% CI) | Baseline (95% CI) | Year 1 of Policy Absolute difference (95% CI) | Year 2 of Policy Absolute difference (95% CI) | Year 3 of Policy Absolute difference (95% CI) |
| *School* | | | | | | | | |
| Total sugars (%) | 31.7 (28.8 , 34.5) | 3.1 (-0.9 , 7.0) | -4.0* (-7.9 , -0.1) | -13.8* (-17.7 , -9.9) | 31.7 (28.8 , 34.5) | 2.9 (-0.9 , 6.8) | -3.5 (-7.3 , 0.3) | -13.2* (-17.1 , -9.3) |
| Saturated fats (%) | 6.5 (5.8 , 7.1) | 0.6 (-0.3 , 1.5) | 0.8 (-0.1 , 1.9) | -1.3* (-2.9 , -0.3) | 6.5 (5.8 , 7.1) | 0.7 (-0.2 , 1.6) | 0.9 (-0.1 , 1.9) | -1.2* (-2.1 , -0.3) |
| Sodium (mg/100 kcal) | 58.7 (52.8 , 64.5) | 12.7 (3.1 , 22.4) | 10.2* (0.5 , 19.9) | -11.6* (-20.2 , -2.9) | 58.7 (52.8 , 64.5) | 11.8 (2.3 , 21.3) | 9.2 (-0.4 , 18.8) | -12.9* (-21.4 , -4.4) |
| *Home* | | | | | | | | |
| Total sugars (%) | 28.0 (26.7 , 29.3) | 0.1 (-1.9 , 2.1) | -2.1* (-3.9 , -0.2) | -5.1* (-6.9 , -3.3) | 28.0 (26.7 , 29.3) | -0.3 (-2.2 , 1.7) | -2.0* (-3.8 , -0.1) | -4.1* (-5.9 , -2.3) |
| Saturated fats (%) | 9.7 (9.2 , 10.2) | -0.3 (-1.0 , 0.4) | 0.1 (-0.6 , 0.9) | 0.1 (-0.7 , 0.8) | 9.7 (9.2 , 10.2) | -0.3 (-1.0 , 0.4) | 0.1 (-0.6 , 0.8) | 0.0 (-0.7 , 0.7) |
| Sodium (mg/100 kcal) | 128.8 (122.7 , 134.9) | -8.7 (-21.2 , 3.7) | -7.0 (-16.1 , 2.0) | 0.2 (-9.3 , 9.9) | 128.8 (122.7 , 134.9) | -8.3 (-17.5 , 3.2) | -7.7 (-11.0 , 4.9) | -3.7 (-7.6 , 10.2) |
| *Other* | | | | | | | | |
| Total sugars (%) | 10.0 (7.7 , 12.3) | 5.8* (2.1 , 9.5) | 6.9* (3.2 , 10.7) | 6.1* (2.3 , 9.9) | 10.0 (7.7 , 12.3) | 5.7* (2.1 , 9.3) | 6.9* (3.2 , 10.7) | 6.6* (2.9 , 10.2) |
| Saturated fats (%) | 3.3 (2.5 , 4.1) | 1.6* (0.4 , 2.8) | 1.6* (0.4 , 2.7) | 1.4* (0.2 , 2.6) | 3.3 (2.5 , 4.1) | 1.7* (0.5 , 2.9) | 1.5* (0.3 , 2.6) | 1.4* (0.2 , 2.6) |
| Sodium (mg/100 kcal) | 27.2 (20.9 , 33.4) | 22.9* (11.5 , 34.4) | 26.4* (14.9 , 37.9) | 24.0* (13.5 , 34.5) | 27.2 (20.9 , 33.4) | 22.5* (11.9 , 32.9) | 24.4* (12.7 , 36.1) | 24.8* (14.2 , 35.5) |
| *Overall* | | | | | | | | |
| Total sugars (%) | 28.7 (27.9 , 29.7) | -0.8 (-2.1 , 0.4) | -1.7* (-2.9 , -0.5) | -2.6* (-3.9 , -1.3) | 28.7 (27.9 , 29.7) | -0.8 (-2.1 , 0.4) | -1.7* (-2.9 , -0.5) | 28.7 (27.9 , 29.7) |
| Saturated fats (%) | 9.7 (9.4 , 10.1) | -0.0 (-0.5 , 0.5) | 0.2 (-0.3 , 0.6) | 0.4 (-0.1 , 1.0) | 9.7 (9.4 , 10.1) | -0.0 (-0.5 , 0.5) | 0.1 (-0.3 , 0.6) | 9.7 (9.4 , 10.1) |
| Sodium (mg/100 kcal) | 116.9 (112.3 , 121.6) | -3.9 (-11.4 , 3.6) | 0.7 (-6.1 , 7.6) | 4.4 (-2.4 , 11.3) | 116.9 (112.3 , 121.6) | -3.5 (-10.9 , 4.0) | 0.8 (-6.0 , 7.7) | 116.9 (112.3 , 121.6) |

Note: Absolute difference is the difference between each year's mean consumption of nutrients of concern after policy compared to baseline. Estimates were derived from fixed-effects models comparing nutrient's consumption in each year (2017, 2018 and 2019) to consumption at baseline (2016). Models excluded weekend days. Covariates in adjusted models include maternal education level, and child's BMI z-score. Data are from the Food Environment Chilean Cohort (FECHIC). For total sugars and saturated fats, we calculated the percentage of energy that each of these nutrients contributed to the total daily energy consumption at each eating location (home, school and other). For sodium, we estimated the intake of sodium (mg) per 100 kcal. *p<0.005
For school analyses, we additionally controlled for other consumption locations (e.g., if outcome was sugars at school, we controlled for sugars at home and sugar at other locations).

**Table S9. Changes in adolescents' percent of energy from total sugars, saturated fats, and sodium after Chile's LFLA implementation by eating location (only weekdays)**

| | **Adjusted models Adolescents (n = 294)** | | | **Unadjusted models Adolescents (n = 294)** | | |
|---|---|---|---|---|---|---|
| **Year** | Baseline (95% CI) | Year 1 of Policy Absolute difference (95% CI) | Year 2 of Policy Absolute difference (95% CI) | Baseline (95% CI) | Year 1 of Policy Absolute difference (95% CI) | Year 2 of Policy Absolute difference (95% CI) |
| *School* | | | | | | |
| Total sugars (%) | 22.8 (20.5 , 25.2) | -2.4 (-5.6 , 0.8) | -7.3* (-10.8 , -3.9) | 22.8 (20.5 , 25.2) | -2.7 (-5.9 , 0.6) | -7.5* (-10.7 , -4.2) |
| Saturated fats (%) | 8.9 (8.1 , 9.8) | 0.5 (-0.7 , 1.8) | -1.9* (-3.2 , -0.6) | 8.9 (8.1 , 9.8) | 0.4 (-0.8 , 1.6) | -2.1* (-3.3 , -0.9) |
| Sodium (mg/100 kcal) | 82.6 (74.6 , 90.6) | 5.6 (-7.4 , 18.6) | -15.2* (-28.8 , -1.7) | 82.6 (74.6 , 90.6) | 3.5 (-8.5 , 15.5) | -17.3* (-29.4 , -5.2) |
| *Home* | | | | | | |
| Total sugars (%) | 20.6 (19.3 , 21.9) | 0.2 (-1.9 , 2.3) | 0.2 (-2.1 , 2.5) | 20.6 (19.3 , 21.9) | 0.6 (-1.4 , 2.7) | 0.8 (-1.3 , 2.7) |
| Saturated fats (%) | 9.0 (8.5 , 9.5) | 0.4 (-0.4 , 1.3) | -0.1 (-0.9 , 0.6) | 9.0 (8.5 , 9.5) | 0.3 (-0.5 , 1.1) | -0.3 (-1.1 , 0.4) |
| Sodium (mg/100 kcal) | 144.4 (136.2 , 152.6) | -5.1 (-18.1 , 8.0) | 1.2 (-12.4 , 14.8) | 144.4 (136.2 , 152.6) | -5.1 (-17.8 , 7.6) | -0.1 (-13.1 , 12.9) |
| *Other* | | | | | | |
| Total sugars (%) | 9.0 (6.5 , 11.5) | 3.6 (-0.3 , 7.6) | 2.9 (-0.9 , 6.8) | 9.0 (6.5 , 11.5) | 3.7 (-0.2 , 7.6) | 2.9 (-0.5 , 6.5) |
| Saturated fats (%) | 2.5 (1.9 , 3.2) | 1.0 (-0.0 , 2.1) | 1.3* (0.2 , 2.4) | 2.5 (1.9 , 3.2) | 1.0 (-0.1 , 2.1) | 1.1* (0.1 , 2.1) |
| Sodium (mg/100 kcal) | 26.4 (19.3 , 33.5) | 18.4* (6.3 , 30.4) | 24.9* (11.1 , 38.7) | 26.4 (19.3 , 33.5) | 15.7* (4.3 , 27.1) | 19.8* (7.4 , 32.4) |
| *Overall* | | | | | | |
| Total sugars (%) | 21.7 (20.7 , 22.7) | 0.6 (-0.8 , 2.1) | 0.6 (-0.9 , 2.1) | 21.7 (20.7 , 22.7) | 0.9 (-0.5 , 2.4) | 1.0 (-0.4 , 2.5) |
| Saturated fats (%) | 9.6 (9.3 , 10.0) | 0.4 (-0.2 , 0.9) | -0.0 (-0.6 , 0.5) | 9.6 (9.3 , 10.0) | 0.2 (-0.3 , 0.8) | -0.2 (-0.8 , 0.3) |
| Sodium (mg/100 kcal) | 131.0 (125.3 , 136.7) | -2.1 (-10.3 , 6.1) | 6.7 (-2.3 , 15.7) | 131.0 (125.3 , 136.7) | -1.8 (-9.9 , 6.3) | 7.6 (-1.2 , 16.3) |

Note: Absolute difference is the difference between each year's mean consumption of nutrients of concern after policy compared to baseline. Estimates were derived from fixed-effects models comparing nutrient's consumption in each year (2017, 2018 and 2019) to consumption at baseline (2016). Models excluded weekend days. Covariates in adjusted models include maternal education level, and child's BMI z-score. Data are from the Growth Obesity Cohort Study (GOCS). For total sugars and saturated fats, we calculated the percentage of energy that each of these nutrients contributed to the total daily energy consumption at each eating location (home, school and other). For sodium, we estimated the intake of sodium (mg) per 100 kcal. *p<0.005
For school analyses, we additionally controlled for other consumption locations (e.g., if outcome was sugars at school, we controlled for sugars at home and sugar at other locations).

**Table S10. Changes in children's percent of energy from total sugars, saturated fats, and sodium by eating location after Chile's law implementation, 2016-2019 (mixed models)**

| **Outcome/ Eating location** | Total Sugars (%) B (95% CI) (n = 961) | Saturated fats (%) B (95% CI) (n = 961) | Sodium (mg/100 kcal) B (95% CI) (n = 961) |
|---|---|---|---|
| *School* | | | |
| Year after policy | | | |
| 1 | -2.6* (-4.7, -0.5) | -0.2 (-0.7 , 0.3) | 2.2 (-3.1 , 7.5) |
| 2 | -4.0* (-6.1 , -1.8) | 0.6* (0.1 , 1.2) | 5.8* (0.3 , 11.2) |
| 3 | -10.3* (-12.6 , -8.1) | -0.4 (-0.9 , 0.2) | -3.5 (-8.9 , 1.9) |
| Maternal education | | | |
| Less than high school | Ref | Ref | Ref |
| High school | 2.3* (-0.1 , 4.8) | 0.2 (-0.4 , 0.8) | 1.5 (-4.2, 7.4) |
| More than high school | 4.9* (2.4, 7.4) | 0.3 (-0.3 , 0.9) | 4.8 (-0.9 , 10.7) |
| Child BMI z-score | 0.1 (-0.5 , 0.8) | -0.1 (-0.2 , 0.1) | 0.2 (-1.4 , 1.8) |
| Nutrient consumption at home | -0.1* (-0.2 , -0.2) | 0.0 (-0.0 , 0.1) | 0.0* (0.0 , 0.1) |
| Nutrient consumption at other locations | -0.1* (-0.1 , -0.0) | -0.0 (-0.1 , 0.0) | -0.1* (-0.1 , -0.0) |
| *Home* | | | |
| Year after policy | | | |
| 1 | -1.3* (-2.4 , -0.3) | -0.1 (-0.5 , 0.3) | -7.0* (-12.8 , -1.2) |
| 2 | -2.3* (-3.4 , -1.2) | -0.3 (-0.7 , 0.1) | -3.3* (-12.1 , -0.3) |
| 3 | -4.1* (-5.2 , -2.9) | -0.1 (-0.6 , 0.3) | -0.8 (-6.8 , 5.2) |
| Maternal education | | | |
| Less than high school | Ref | Ref | Ref |
| High school | -0.3 (-1.1 , 1.6) | 0.7* (0.2 , 1.2) | -1.7 (-8.4 , 5.0) |
| More than high school | -2.2 (-1.6 , 1.1) | 0.8 * (0.3 , 1.3) | -1.5 (-8.2 , 5.2) |
| Child BMI z-score | 0.0 (-0.3 , 0.4) | -0.1* (-0.3 , -0.0) | 3.0* (1.1 , 4.8) |
| Weekday | -0.0 (-1.2 , 1.2) | -0.5* (-0.9 , -0.1) | -1.8 (-8.1 , 4.4) |
| Nutrient consumption at school | -0.0* (-0.0 , -0.0) | 0.0* (0.0 , 0.0) | 0.0* (0.0 , 0.1) |
| Nutrient consumption at other locations | 0.0 (-0.0 , 0.0) | -0.0 (-0.0 , 0.0) | -0.1* (-0.1 , -0.0) |
| *Other* | | | |
| Year after policy | | | |
| 1 | 2.8* (0.7 , 4.8) | 1.3* (0.6 , 1.9) | 13.5* (6.5 , 20.5) |
| 2 | 3.7* (1.6 , 5.9) | 1.3* (0.6 , 1.9) | 14.4* (7.2 , 21.6) |
| 3 | 2.9* (0.8 , 5.4) | 1.4 *(0.7 , 2.1) | 14.9* (7.7 , 22.2) |
| Maternal education | | | |
| Less than high school | Ref | Ref | Ref |
| High school | 1.3 (-0.9 , 3.5) | 0.9* (0.2 , 1.7) | 11.9* (3.8 , 20.0) |
| More than high school | 2.4* (0.1 , 4.7) | 1.2* (0.5 , 2.0) | 10.3* (2.1 , 18.4) |
| Child BMI z-score | -0.1 (-0.7 , 0.6) | -0.0 (-0.2 , 0.2) | 0.8 (-1.3 , 3.1) |
| Weekday | -2.6* (-4.8 , -0.4) | -1.1* (-1.8 , -0.4) | -23.4* (-30.9, -15.9) |
| Nutrient consumption at school | -0.0* (-0.1 , -0.0) | -0.0 (-0.1 , 0.0) | -0.0 (-0.1 , 0.0) |
| Nutrient consumption at home | 0.0 (-0.0 , 0.1) | -0.0 (-0.1 , 0.0) | -0.1* (-0.1 , -0.0) |
| *Overall* | | | |
| Year after policy | | | |
| 1 | -1.6* (-2.3 , -0.9) | 0.1 (-0.2 , 0.4) | -4.2* (-8.2 , -0.2) |
| 2 | -1.9* (-2.7 , -1.2) | 0.1 (-0.2 , 0.4) | 0.7 (-6.0 , 2.1) |
| 3 | -2.8* (-3.6, -2.1) | 0.4* (-0.2 , 0.8) | 5.4* (-0.2 , 8.0) |

| | | | |
|---|---|---|---|
| Maternal education | | | |
| Less than high school | Ref | Ref | Ref |
| High school | 0.2 (-0.6 , 1.2) | 0.4* (0.0 , 0.8) | 0.12 (-4.5 , 4.7) |
| More than high school | 0.2 (-0.7 , 1.2) | 0.5* (0.2 , 0.9) | 0.9 (-3.7 , 5.6) |
| Child BMI z-score | -0.1 (-1.0 , 1.4) | -0.1* (-0.2 , -0.0) | 1.9* (0.6 , 3.2) |
| Weekday | 0.5 (-0.2 , 1.3) | -0.5* (-0.8 , -0.2) | -10.6* (-14.6 , -6.7) |

Note: Estimates were derived from mixed models comparing nutrient's consumption in each year (2017, 2018 and 2019) to consumption at baseline (2016). Data are from the Food Environment Chilean Cohort (FECHIC). For total sugars and saturated fats, we calculated the percentage of energy that each of these nutrients contributed to the total daily energy consumption at each eating location (home, school and other). For sodium, we estimated the intake of sodium (mg) per 100 kcal. Consumption includes weekends. *p<0.005; confidence intervals in parenthesis.

**Table S11. Changes in adolescents' percent of energy from total sugars, saturated fats, and sodium by eating location after Chile's law implementation with covariate coefficients, 2016-2018 (mixed models)**

| **Outcome/ Eating location** | Total Sugars (%) B (95% CI) (n = 963) | Saturated fats (%) B (95% CI) (n = 963) | Sodium (mg/100 kcal) B (95% CI) (n = 963) |
|---|---|---|---|
| *School* | | | |
| Year after policy | | | |
| 1 | -1.8* (-3.6 , -0.1) | -0.9* (-1.6 , -0.3) | 0.0 (-6.3 , 6.4) |
| 2 | -4.1* (-5.8 , -2.4) | -1.7* (-2.3 , -1.0) | -5.1 (-11.3 , 1.2) |
| Maternal education | | | |
| Less than high school | Ref | Ref | Ref |
| High school | 0.1 (-1.6 , 1.9) | 0.2 (-0.4 , 0.8) | 1.3 (-4.9 , 7.6) |
| More than high school | 0.5 (-1.5 , 2.6) | -0.0 (-0.7 , 0.7) | 2.5 (-4.7 , 9.7) |
| Adolescent BMI z-score | -0.3 (-0.9 , 0.3) | -0.3* (-0.5 , -0.1) | -1.2 (-3.5 , 1.1) |
| Nutrient consumption at home | 0.0 (-0.0 , 0.1) | 0.0 (-0.0 , 0.1) | 0.0 (-0.0 , 0.1) |
| Nutrient consumption at other locations | -0.0 (-0.1 , 0.0) | -0.1* (-0.1 , -0.0) | -0.0 (-0.0 , 0.0) |
| *Home* | | | |
| Year after policy | | | |
| 1 | 0.8 (-0.3 , 2.1) | 0.3 (-0.1 , 0.8) | 1.9 (-5.5 , 9.4) |
| 2 | 0.5 (-0.7 , 1.7) | -0.1 (-0.6 , 0.3) | 1.6 (-5.8 , 8.9) |
| Maternal education | | | |
| Less than high school | Ref | Ref | Ref |
| High school | -0.3 (-1.6 , 0.9) | 0.0 (-0.4 , 0.4) | -1.0 (-8.5 , 6.5) |
| More than high school | -0.5 (-1.9 , 0.9) | 0.5* (0.0 , 1.1) | 2.1 (-6.5 , 10.7) |
| Adolescent BMI z-score | -0.8* (-1.3 , -0.4) | 0.0 (-0.1 , 0.2) | 5.2* (2.4 , 7.9) |
| Weekday | 0.2 (-1.2 , 1.2) | -0.0 (-0.6 , 0.5) | 6.3 (-2.6 , 15.1) |
| Nutrient consumption at school | 0.0 (-0.0 , 0.0) | 0.0 (-0.0 , 0.0) | 0.0 (-0.0 , 0.1) |
| Nutrient consumption at other locations | -0.0 (-0.0 , 0.0) | -0.0* (-0.1 , -0.0) | -0.0 (-0.0 , 0.0) |
| *Other* | | | |
| Year after policy | | | |
| 1 | 3.0* (0.9 , 5.1) | 0.8* (0.1 , 1.4) | -14.8 (-136.1 , 106.5) |
| 2 | 3.1* (1.0 , 5.1) | 0.5* (-0.1 , 1.1) | 41.8 (-77.7 – 161.4) |
| Maternal education | | | |
| Less than high school | Ref | Ref | Ref |
| High school | 1.0 (-1.1 , 3.1) | 0.3 (-0.3 , 0.9) | 23.8 (-91.7 , 139.3) |
| More than high school | -2.0 (-0.4 , 4.5) | 0.9 (0.2 , 1.6) | 91.7 (-40.7 , 224.2) |
| Adolescent BMI z-score | 0.3 (-0.5 , 1.1) | -0.0 (-0.3 , 0.2) | 25.9 (-16.3 , 68.3) |
| Weekday | -3.8* (-6.3 , -1.4) | -1.6* (-2.4 , -0.9) | 26.1 (-114.9 , 167.1) |
| Nutrient consumption at school | 0.0 (-0.0 , 0.1) | -0.0 (-0.1 , 0.0) | -0.3 (-1.1 , 0.5) |
| Nutrient consumption at home | -0.0 (-0.1 , 0.1) | -0.0* (-0.1 , -0.0) | -0.3 (-0.9 , 0.3) |
| *Overall* | | | |
| Year after policy | | | |
| 1 | 0.8* (0.0 , 1.6) | 0.0 (-0.3 , 0.3) | 4.5 (-0.5 , 9.5) |
| 2 | 0.5 (-0.3 , 1.3) | -0.2 (-0.5 , 0.1) | 8.1* (3.1 , 13.0) |
| Maternal education | | | |
| Less than high school | Ref | Ref | Ref |

| | | | |
|---|---|---|---|
| High school | 0.2 (-0.7 , 1.1) | 0.3 (-0.0 , 0.6) | -0.3 (-5.3 , 4.7) |
| More than high school | 0.2 (-0.7 , 1.2) | 0.7* (0.3 , 1.1) | 2.1 (-3.7 , 7.8) |
| Adolescent BMI z-score | -0.4* (-0.7 , -0.1) | -0.0 (-0.1 , 0.1) | 3.9* (2.1 , 5.8) |
| Weekday | -0.0 (-0.9 , 0.9) | -0.1 (-0.5 , 0.2) | -6.3* (-11.8 , -0.8) |

Note: Estimates were derived from mixed effects models comparing nutrient's consumption in each year (2017 and 2018) to consumption at baseline (2016). Data are from the Growth Obesity Cohort Study (GOCS). For total sugars and saturated fats, we calculated the percentage of energy that each of these nutrients contributed to the total daily energy consumption at each eating location (home, school and other). For sodium, we estimated the intake of sodium (mg) per 100 kcal. Consumption includes weekends. *p<0.005; confidence intervals in parenthesis.
