# Supplementary material for: Changes in children’s and adolescents’ dietary intake after the implementation of Chile’s law of food labeling, advertising and sales in schools: a longitudinal study
Source: Int J Behav Nutr Phys Act. 2023 Apr 4;20:40. doi: 10.1186/s12966-023-01445-x (PMC10074676; doi:10.1186/s12966-023-01445-x)
Supplement: Supplementary file 2 — Additional File 2. STROBE Statement Checklist of items that should be included in reports of observational studies. [file 12966_2023_1445_MOESM2_ESM.pdf]

Additional File 2. STROBE Statement—Checklist of items that should be included in reports of ***observational studies.***

Item	#	Recommendation	STROBE-nut[1]	Reported
**Title and abstract**	1	(*a*) Indicate the study's design with a commonly used term in the title or the abstract	nut-1. State the dietary/nutritional assessment method(s) used in the title, abstract, or keywords	Title, Background and Methods sections of abstract.
		(*b*) Provide in the abstract an informative and balanced summary of what was done and what was found		Abstract
**Introduction**				
Background/rationale	2	Explain the scientific background and rationale for the investigation being reported		Page 3 - 5
Objectives	3	State specific objectives, including any prespecified hypotheses		Page 5, lines 103-107
**Methods**				
Study design	4	Present key elements of study design early in the paper		Page 5, 1st paragraph (lines 110-113) in Methods.
Setting	5	Describe the setting, locations, and relevant dates, including periods of recruitment, exposure, follow-up, and data collection	nut-5. Describe any characteristics of the study settings that might affect the dietary intake or nutritional status of the participants, if applicable.	Page 5-6, 1st and 2nd paragraphs in Methods.
Participants	6	(*a*) Give the eligibility criteria, and the sources and methods of selection of participants	nut-6. Report particular dietary, physiological, or nutritional characteristics that were considered when selecting the target population.	Page 5-6, 1st and 2nd paragraphs in Methods. Reference to additional studies describing eligibility criteria.
Variables	7	Clearly define all outcomes, exposures, predictors, potential confounders, and effect modifiers. Give diagnostic criteria, if applicable	nut-7.1. Clearly define foods, food groups, nutrients, or other food components.<br>nut-7.2. When using dietary patterns or indices, describe the methods to obtain them and their nutritional properties.	Page 6-8. "Data collection" (dietary data, outcomes and covariates).
Data sources/ measurement	8*	For each variable of interest, give sources of data and details of methods of assessment (measurement). Describe comparability of assessment methods if there is more than one group.	nut-8.1. Describe the dietary assessment method(s), e.g., portion size estimation, number of days and items recorded, how it was developed and administered, and how quality was assured. Report if and how supplement intake was assessed.	Page 6-7. Specifically, "Data collection" section.
			nut-8.2. Describe and justify food composition data used. Explain the procedure to match food composition with consumption data. Describe the use of conversion factors, if applicable.	Page 6. First paragraph in Data collection. Lines 136-143.

			nut-8.3. Describe the nutrient requirements, recommendations, or dietary guidelines and the evaluation approach used to compare intake with the dietary reference values, if applicable.	NA. We do not compare with dietary reference values.
			nut-8.4. When using nutritional biomarkers, additionally use the STROBE Extension for Molecular Epidemiology (STROBE-ME). Report the type of biomarkers used and their usefulness as dietary exposure markers.	NA.
			nut-8.5. Describe the assessment of non-dietary data (e.g., nutritional status and influencing factors) and timing of the assessment of these variables in relation to dietary assessment.	Page 7. "Data Collection", lines 153-159.
			nut-8.6. Report on the validity of the dietary or nutritional assessment methods and any internal or external validation used in the study, if applicable.	NA. No validation study was used.
Bias	9	Describe any efforts to address potential sources of bias	nut-9. Report how bias in dietary or nutritional assessment was addressed, e.g., misreporting, changes in habits as a result of being measured, or data imputation from other sources.	Page 9 under sensitivity analyses, we repeated analyses excluding implausible values and considering participants who did not have all study years (pooled analysis, mixed models). Line 204-207.
Study size	10	Explain how the study size was arrived at		Page 6. Study population and setting.
Quantitative variables	11	Explain how quantitative variables were handled in the analyses. If applicable, describe which groupings were chosen and why	nut-11. Explain the categorization of dietary/nutritional data (e.g., use of N-tiles and handling of non-consumers) and the choice of reference category, if applicable.	Page 8-9. Statistical Analyses.
Statistical methods	12	(*a*) Describe all statistical methods, including those used to control for confounding	nut-12.1. Describe any statistical method used to combine dietary or nutritional data, if applicable.	Page 8-9. Statistical Analyses.
		(*b*) Describe any methods used to examine subgroups and interactions	nut-12.2. Describe and justify the method for energy adjustments, intake modeling, and use of weighting factors, if applicable.	Page 8-9. Statistical Analyses.
		(*c*) Explain how missing data were addressed	nut-12.3. Report any adjustments for measurement error, i.e., from a validity or calibration study.	Page 6. Study population and setting. Lines 122-131.
		(*d*) If applicable, describe analytical methods taking account of sampling strategy		NA

		(*e*) Describe any sensitivity analyses		Page 9-10. Statistical Analyses.
**Results**				
Participants	13*	(a) Report numbers of individuals at each stage of study—eg numbers potentially eligible, examined for eligibility, confirmed eligible, included in the study, completing follow-up, and analysed	nut-13. Report the number of individuals excluded based on missing, incomplete, or implausible dietary/nutritional data.	Page 6. Study population and setting. Lines 122-131; Page 10-11. Results
		(b) Give reasons for non-participation at each stage		NA
		(c) Consider use of a flow diagram		Additional file 1. Figure S1 and S2.
Descriptive data	14*	(a) Give characteristics of study participants (eg demographic, clinical, social) and information on exposures and potential confounders	nut-14. Give the distribution of participant characteristics across the exposure variables if applicable. Specify if food consumption of total population or consumers only were used to obtain results.	**Table 1** presents characteristics of study participants.
		(b) Indicate number of participants with missing data for each variable of interest		
Outcome data	15*	Report numbers of outcome events or summary measures		NA
Main results	16	(*a*) Give unadjusted estimates and, if applicable, confounder-adjusted estimates and their precision (eg, 95% confidence interval). Make clear which confounders were adjusted for and why they were included	nut-16. Specify if nutrient intakes are reported with or without inclusion of dietary supplement intake, if applicable.	For table simplicity, only adjusted and unadjusted outcome estimates are presented (Table 2 and Table 3). Covariate coefficients can be found in Additional File 1. Table S4 and S5.
		(*b*) Report category boundaries when continuous variables were categorized		NA
		(*c*) If relevant, consider translating estimates of relative risk into absolute risk for a meaningful time period		NA
Other analyses	17	Report other analyses done—eg analyses of subgroups and interactions, and sensitivity analyses	nut-17. Report any sensitivity analysis (e.g., exclusion of misreporters or outliers) and data imputation, if applicable.	Page 13-14. Lines 276-297. Additional file 1. Table S6 to Table S11.
**Discussion**				
Key results	18	Summarise key results with reference to study objectives		Page 14. First paragraph of Discussion.
Limitations	19	Discuss limitations of the study, taking into account sources of potential bias or imprecision. Discuss both direction and magnitude of any potential bias	nut-19. Describe the main limitations of the data sources and assessment methods used and implications for the interpretation of the findings.	Page 17.

Interpretation	20	Give a cautious overall interpretation of results considering objectives, limitations, multiplicity of analyses, results from similar studies, and other relevant evidence	nut-20. Report the nutritional relevance of the findings, given the complexity of diet or nutrition as an exposure.	Page 14-17.
Generalisability	21	Discuss the generalisability (external validity) of the study results		Page 17. Limitations in Discussion.
**Other information**				
Funding	22	Give the source of funding and the role of the funders for the present study and, if applicable, for the original study on which the present article is based	nut-22.1. Describe the procedure for consent and study approval from ethics committee(s).	Page 19. “Funding” and “Ethics approval and consent to participate”.
			nut-22.2. Provide data collection tools and data as online material or explain how they can be accessed.	Page 19. “Availability of data and material”.

[1] Updated to include guidelines from: Lachat C, Hawwash D, Ocké MC, Berg C, Forsum E, Hörnell A, Larsson CL, Sonestedt E, Wirfält E, Åkesson A, Kolsteren P. Strengthening the Reporting of Observational Studies in Epidemiology–nutritional epidemiology (STROBE-nut): An extension of the STROBE statement. Nutrition bulletin. 2016 Sep;41(3):240-51. https://journals.plos.org/plosmedicine/article?id=10.1371/journal.pmed.1002036

*Give information separately for exposed and unexposed groups.

Note: An Explanation and Elaboration article discusses each checklist item and gives methodological background and published examples of transparent reporting. The STROBE checklist is best used in conjunction with this article (freely available on the Web sites of PLoS Medicine at http://www.plosmedicine.org/, Annals of Internal Medicine at http://www.annals.org/, and Epidemiology at http://www.epidem.com/). Information on the STROBE Initiative is available at www.strobe-statement.org.
